# Stable insular spectral patterns underlie dynamic pain encoding in chronic neuropathic pain

**DOI:** 10.1093/braincomms/fcag299

**Published:** 2026-08-03

**Authors:** Chang-Chia Liu, Mark Quigg, Patrick H Finan, Shayan Moosa, W Jeffrey Elias

**Affiliations:** Department of Neurological Surgery, University of Virginia School of Medicine, Charlottesville, VA 22908, USA; Department of Neurology, University of Virginia School of Medicine, Charlottesville, VA 22908, USA; Department of Anesthesiology, University of Virginia School of Medicine, Charlottesville, VA 22908, USA; Department of Neurological Surgery, University of Virginia School of Medicine, Charlottesville, VA 22908, USA; Department of Neurological Surgery, University of Virginia School of Medicine, Charlottesville, VA 22908, USA

**Keywords:** Insula, Chronic neuropathic pain, Intracranial EEG, Pain intensity, Pain unpleasantness

## Abstract

Chronic neuropathic pain persists for years yet fluctuates markedly from day to day, raising a fundamental question: how do stable neural patterns relate to dynamic pain experience? The insula is a central hub for pain processing, but it remains unclear whether ongoing pain is associated with enduring baseline neural patterns within the insula or day-to-day variation in neural activity.

We analysed multiday bilateral intracranial electroencephalography recordings from six individuals with chronic unilateral neuropathic pain, comprising 34 days of resting-state data. Spectral power, peak-derived spectral metrics and intra-insular functional connectivity (coherence and phase-locking value) were quantified across anterior and posterior insular subregions. Linear mixed-effects models were used to distinguish stable within-cohort neural patterns from within-subject, day-to-day pain-related fluctuations in pain intensity and unpleasantness, with explicit control for longitudinal temporal drift.

The insula exhibited stable within-cohort spectral organization characterized by an anterior–posterior gradient with lower posterior fast-band power and spectral slowing, together with greater posterior spectral power in the hemisphere contralateral to the chronic pain side. Superimposed on this stable pattern, daily pain intensity was most strongly associated with increased contralateral posterior alpha power, accounting for approximately 33% of within-subject variance. Pain unpleasantness showed its strongest association with increased contralateral posterior beta power, accounting for approximately 19% of within-subject variance. Peak-derived spectral metrics and intra-insular connectivity largely reflected stable baseline patterns, with comparatively modest day-to-day pain-related variation.

These exploratory findings suggest that chronic neuropathic pain is associated with stable insular spectral patterns upon which frequency-specific oscillatory variation relates to daily pain experience. Posterior insular alpha and beta oscillations showed partially distinct associations with pain intensity and unpleasantness, respectively, whereas intra-insular connectivity primarily reflected stable network structure. Posterior insular spectral power may therefore provide a candidate physiological signal for tracking pain states.

## Teaser

Liu *et al*. report multiday bilateral intracranial EEG recordings in chronic neuropathic pain. Stable insular spectral organization was observed across days, while posterior insular alpha and beta rhythms showed partially distinct associations with pain intensity and unpleasantness, supporting spectral power as a candidate pain-state signal.

## Graphical Abstract

**Graphical Abstract:**
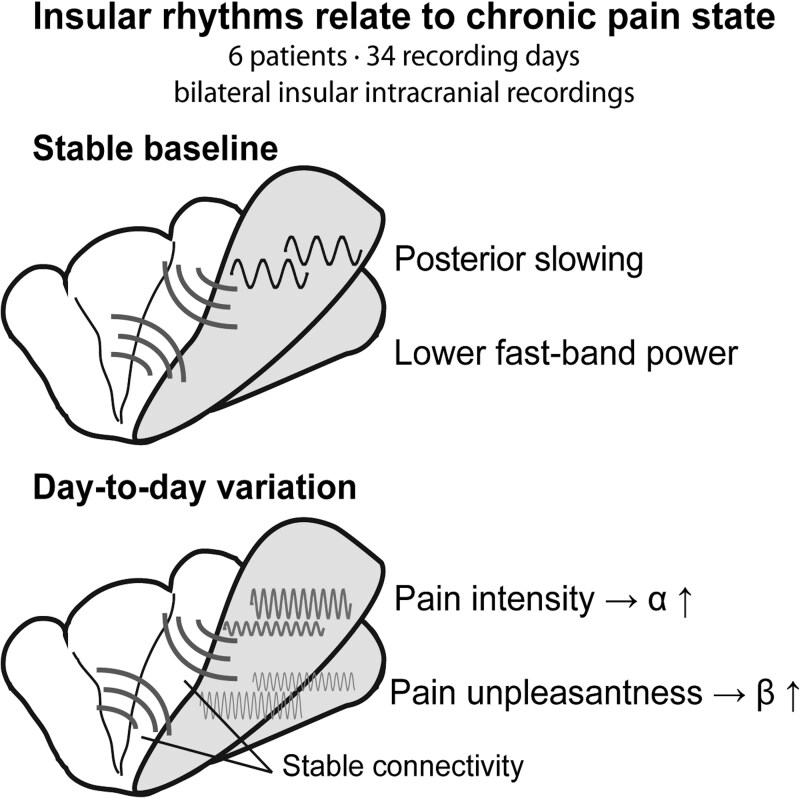

## Introduction

Chronic neuropathic pain is not static: it persists for years yet fluctuates from day to day, implying a combination of enduring disease-related neural alterations and day-to-day pain-related variation. Distinguishing these components is essential for mechanistic models and for translational efforts to develop physiologically grounded sensing biomarkers and closed-loop neuromodulation. The insular cortex is a central hub in pain processing. It receives dense nociceptive and interoceptive inputs from thalamic and brainstem pathways, integrates multimodal information and contributes to both sensory–discriminative and affective–motivational dimensions of pain.^1-3^ Neuroimaging studies consistently implicate the insula in experimental and clinical pain,^4,5^ yet its intrinsic electrophysiological organization during spontaneous ongoing pain remains poorly understood.

Human intracranial recordings provide a rare window into insular physiology with millisecond precision and high spatial specificity. However, pain-related intracranial studies have typically been constrained by unilateral sampling (a key limitation given the lateralized nature of nociceptive pathways), single-day recordings or task-evoked paradigms that conflate intrinsic activity with stimulus-driven responses.^6-10^ These constraints limit the ability to disentangle stable baseline insular spectral patterns from day-to-day pain-related associations observed during chronic pain. Two fundamental questions therefore remain unresolved: (i) what stable electrophysiological patterns characterize spontaneous insular activity in chronic neuropathic pain, and (ii) how do daily fluctuations in pain intensity and pain unpleasantness relate to local rhythms and intra-insular network coupling?

Contemporary frameworks of chronic pain have proposed altered oscillatory operating modes, including spectral slowing and changes in alpha–beta activity. These models provide interpretive context, although the present recordings do not directly test thalamic mechanisms.^11,12^ At the same time, converging evidence from imaging, stimulation and lesion studies suggests that sensory and affective components of pain may be supported by partially differentiated anterior and posterior insular subregions.^13-15^ Yet electrophysiological markers that relate to naturalistic, day-to-day fluctuations in pain dimensions under resting-state conditions have not been clearly identified, limiting progress towards objective sensing signals and principled neuromodulatory targets.

To address these gaps, we analysed multiday, bilateral intracranial EEG recordings obtained from individuals undergoing insular depth electrode implantation as part of a clinical trial of deep brain stimulation (DBS) for chronic neuropathic pain. Resting-state recordings were acquired under standardized conditions across several days, enabling us to distinguish neural patterns that were stable across days from within-subject, day-to-day pain-related variation. Using bipolar signals and linear mixed-effects modelling with explicit control for longitudinal temporal drift, we quantified spectral power, peak-derived spectral metrics and intra-insular functional connectivity across anterior and posterior insular subregions in relation to daily ratings of pain intensity and unpleasantness.

We hypothesized that chronic pain would be characterized by a stable baseline pattern of insular physiology, with day-to-day pain fluctuations reflected more strongly in local spectral power than in large-scale connectivity reorganization.

## Materials and methods

### Study participants

Six adults with medically refractory chronic neuropathic pain were enrolled in a staged, FDA-monitored clinical trial of insular neuromodulation (NCT05404581; FDA IDE G200049; Table 1). The study protocol was approved by the University of Virginia Institutional Review Board (protocol number: HSR200117) and conducted in accordance with the Declaration of Helsinki. All participants underwent and passed comprehensive multidisciplinary clinical evaluations to confirm candidacy for DBS implantation prior to enrolment. Written informed consent was obtained for all study procedures. Eligibility required neuropathic pain duration >3 years, inadequate response to conventional management and willingness to undergo bilateral insular depth electrode implantation. The cohort presented with diverse aetiologies of chronic unilateral neuropathic pain, primarily involving central post-stroke, facial trigeminal and spinal origins (Supplementary Table 1). Resting-state iEEG was analysed from designated non-stimulation intervals during the parent trial, acquired at a consistent time of day and >30 min post-stimulation to minimize transient physiological effects. At study enrolment, baseline pain intensity ranged from severe (>6/10) to very severe (up to 10/10), with substantial functional impairment despite multidisciplinary care. All participants completed a 7-day inpatient monitoring period during which the electrophysiological recordings analysed in this study were obtained. Pain and other centrally active medications were kept stable throughout the monitoring period to minimize acute pharmacokinetic confounding of day-to-day electrophysiological signals.

**Table 1:** Participant demographics and clinical characteristics

Patient ID	Age (yrs)	Sex	Pain side	Pain duration (yrs)	Primary pain diagnosis	Key clinical history
001	79	F	Left	10	Postsurgical lower-extremity neuropathic pain	Severe left lower-extremity neuropathic pain after multiple lumbar and thoracolumbar spine surgeries
002	69	F	Left	10	Trigeminal neuropathic pain	Severe left facial neuropathic pain involving V1–V3 and scalp, refractory to multiple prior interventions
003	68	M	Left	6	Central post-stroke pain	Left hemibody and facial central neuropathic pain after right thalamic infarct
004	43	F	Right	16	Central post-stroke pain	Right hemibody burning pain after lateral medullary infarct
005	59	F	Right	8	Trigeminal anaesthesia dolorosa	Severe right facial neuropathic pain after petroclival meningioma surgery
006	70	M	Left	3	Thalamic pain syndrome	Left hemibody neuropathic pain after right thalamic stroke

Note: ‘yrs’ = years. All participants had failed conventional medical management prior to enrolment.

### Trans-insular depth electrode implantation

**Figure 1:**
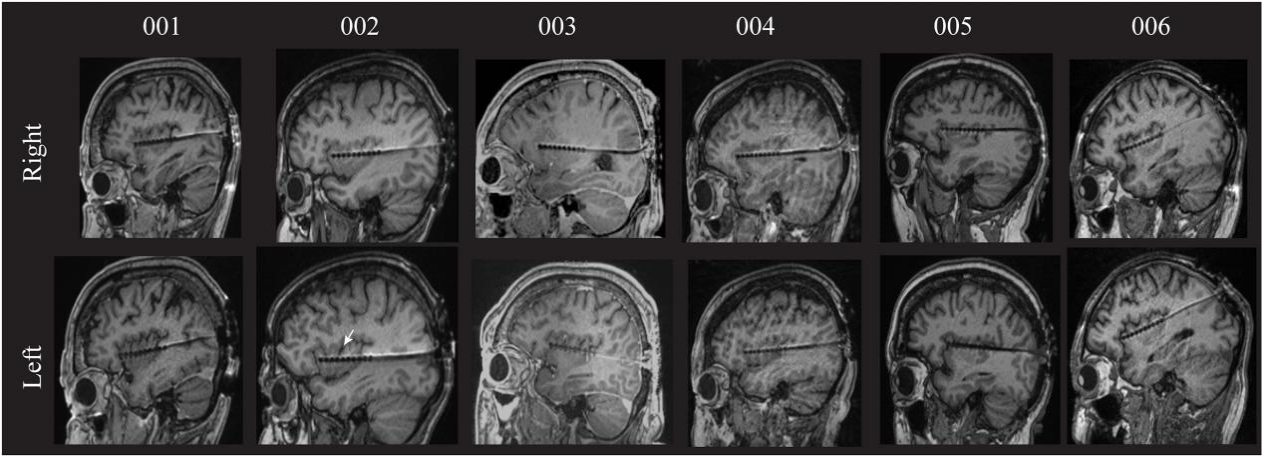
**Localization of bilateral insular depth electrodes.** T1-weighted MRI sections illustrate the locations of depth electrode contacts within the insular cortex for all six participants. Top row: right hemisphere; bottom row: left hemisphere. Columns correspond to individual participants (001–006). Electrode contacts span anterior and posterior insular subregions, providing bilateral coverage for subsequent spectral and connectivity analyses.

All participants underwent stereotactic bilateral trans-insular depth electrode implantation (Fig. 1). Each hemisphere received one 10-contact Ad-Tech depth electrode (SD10R-SP05X-000) with 5 mm centre-to-centre spacing, providing rostrocaudal sampling of the insula. Trajectories were stereotactically planned and advanced through the frontal operculum into the insula along a longitudinal anterior-to-posterior path, permitting simultaneous recording from anterior and posterior insular subregions in both hemispheres.

### Insular subregion labelling

Electrode localization and anatomical labelling were performed entirely in native space using postoperative high-resolution structural MRI. Contact positions were identified manually using multiplanar reconstruction in a quadrant view, enabling concurrent inspection of axial, coronal, sagittal and rendered 3D volumes. This approach provides high spatial fidelity and avoids inaccuracies inherent in automated coordinate-based methods. Insular boundaries were delineated slice-by-slice, using the central sulcus of the insula as the boundary between anterior and posterior subregions. Contacts located directly within the central sulcus were excluded from analysis to ensure distinct segregation between anterior and posterior subregions. While MNI coordinates were extracted for reference and documentation (Supplementary Table 2), the final anatomical assignment relied exclusively on direct visual inspection to ensure individual precision. All bipolar channels were harmonized to a consistent anatomical labelling scheme.

### Intracranial EEG acquisition and preprocessing

Resting-state intracranial EEG was recorded daily at a consistent morning time point, and participants lay quietly with eyes open for a 5-min acquisition to minimize circadian and arousal-related variability. Signals were recorded at 4096 Hz using a Natus Quantum amplifier, downsampled to 1000 Hz, band-pass filtered (0.1–300 Hz) and denoised using a 58–62 Hz notch filter. Recordings were visually inspected daily to identify contacts with excessive noise, persistent artefacts or flat channels. Spectral outlier detection, followed by visual confirmation, was used to generate a daily bad-channel list. Bipolar channels were constructed from adjacent contacts within each insular subregion, producing 10–14 potential bipolar pairs per participant per day (Supplementary Table 3). Bipolar pairs involving any excluded monopolar contact were removed. Only artefact-free bipolar channels were included in subsequent analyses.

### Spectral and functional connectivity analysis

#### Spectral analysis

Power spectral density (PSD) was estimated using Welch’s method. Continuous data were segmented into non-overlapping 5-s epochs. For each epoch, PSD estimates were computed using a 2048-point Hamming window with 50% overlap and an 8192-point fast Fourier transform (FFT). Frequencies from 0 to 55 Hz were retained. Power was evaluated as log-transformed power expressed in dB and relative power within canonical frequency bands: delta (1–4 Hz), theta (4–8 Hz), alpha (8–13 Hz), beta (13–30 Hz) and gamma (30–55 Hz). Peak-derived features were extracted from the same spectra. Peak dominant frequency (PDF) was defined as the frequency of maximal spectral amplitude within the 5–15 Hz range, and peak dominant power (PDP) was the amplitude at that frequency. The Slow Index, calculated as the theta-to-alpha ratio, quantified low-frequency dominance. As a sensitivity analysis, spectral components were decomposed into aperiodic and residual periodic elements to test whether band-limited pain-related effects could be explained by broadband spectral slope; full details are provided in the Supplementary Statistical Methods.

#### Connectivity analysis

Functional connectivity was quantified using two complementary metrics: magnitude-squared coherence (COH) and phase-locking value (PLV). Coherence spectra were computed for each bipolar channel pair using the same 5-s epochs, and band-limited coherence values were obtained by averaging within canonical frequency ranges. Phase-locking value was used to estimate phase synchrony independent of amplitude covariance. For each epoch, signals were band-pass filtered into canonical bands, and the instantaneous analytic phase was extracted using the Hilbert transform. Phase-locking value was computed as the circular mean of phase differences between channel pairs. Epoch-level spectral and connectivity metrics were subsequently aggregated to channel–day and pair–day values, respectively, before mixed-effects modelling.

### Pain intensity and unpleasantness assessment

Immediately before each resting-state EEG recording, participants rated their chronic pain intensity and chronic pain unpleasantness on 11-point numerical rating scales from 0 to 10. Intensity and unpleasantness ratings were collected separately using identical scales. Participants rated the average intensity of their chronic pain over the preceding hour, limited to the chronically affected side. Unpleasantness was rated using the prompt, ‘How unpleasant do you feel about your chronic pain?’ These ratings served as behavioural regressors in the day-to-day pain-related mixed-effects models (Supplementary Table 4).

### Statistical analysis

Spectral power, peak-derived metrics, coherence and phase-locking value were analysed using linear mixed-effects models implemented in MATLAB R2024b (fitlme; MathWorks, Natick, MA). Metrics were first calculated at the epoch level and then aggregated to channel–day values for spectral measures and pair–day values for connectivity before mixed-effects modelling, thereby avoiding pseudo-replication. Spectral power was modelled in decibels, coherence values were Fisher Z-transformed, and phase-locking values were logit-transformed prior to modelling. Laterality was defined relative to each participant’s chronic pain side (ipsilateral versus contralateral).

Analyses followed a two-stage framework designed to first characterize stable baseline organization of insular physiology and then examine how day-to-day fluctuations in pain relate to deviations from those baselines.

In the first stage, linear mixed-effects models were used to quantify stable baseline organization of insular spectral power and functional connectivity across anatomical subregions (anterior versus posterior; ipsilateral versus contralateral). Because systematic temporal drift in spectral power was observed across recording days, all models explicitly incorporated a subject-centred day index (Day_Index_c) as a fixed effect to model and account for longitudinal temporal drift. This approach ensured that stable anatomical organization was estimated independently of slow recording-related settling, inpatient adaptation or other non-pain-related longitudinal changes, thereby isolating reproducible spatial features of insular physiology independent of daily pain fluctuations. Day_Index_c therefore captures across-day drift within subject rather than within-recording variability.

In the second stage, daily pain ratings were incorporated as within-subject predictors to test whether fluctuations in pain intensity or pain unpleasantness were associated with systematic day-level deviations from each participant’s baseline physiology. Pain ratings were centred within-subject so that estimated effects reflect within-individual changes relative to each participant’s typical pain level, rather than between-subject differences. Pain intensity and unpleasantness were evaluated in separate parallel model families to avoid multicollinearity.

Functional connectivity analyses were conducted separately for coherence and phase-locking values and stratified by insular subregion, given known differences in baseline coupling across within-anterior, within-posterior and anterior–posterior channel pairs. Fixed-effect significance was assessed using t-statistics derived from the fitted mixed-effects models, with multiple comparisons controlled using the Benjamini–Hochberg false discovery rate procedure within predefined model families. Effect sizes are reported as partial R^2^. Full model specifications, correction procedures and sensitivity analyses are provided in the Supplementary Statistical Methods.

## Results

### Overview of dataset and modelling framework

Intracranial EEG was analysed from six participants with chronic unilateral neuropathic pain who contributed 34 days of resting-state recordings (range: four to seven per participant). After quality inspection, 264 bipolar channel–day observations were retained for spectral analyses, with pair–day observations used for connectivity analyses. Across retained recordings, 15 840 artefact-free epochs were available and summarized at the channel–day and pair–day levels. Bipolar channels were assigned to four insular quadrants (anterior/posterior × ipsilateral/contralateral relative to the pain side), enabling anatomically resolved analyses of local oscillatory activity and intra-insular connectivity (Supplementary Table 2). Throughout the Results, laterality is defined relative to each participant’s chronic pain side, and posterior insular power is described relative to anterior insular power within the same hemisphere.

During the inpatient monitoring period, daily pre-recording pain ratings varied substantially within participants for pain intensity (mean ± SD: 5.41 ± 2.30) and unpleasantness (4.85 ± 2.33), with all participants varying by at least two points across recording days (Supplementary Table 4). Spectral power showed systematic temporal drift across monitoring days (e.g. contralateral posterior theta: *β* ≈ −0.77 dB/day, 95% CI −1.01 to −0.53; Supplementary Table 10). Accordingly, all mixed-effects models included a subject-centred Day_Index_c covariate, allowing pain-related associations to be estimated independently of slow longitudinal changes.

Results are organized into two outcome domains: spectral power and intra-insular connectivity. Within each domain, we report stable baseline patterns followed by day-to-day pain-related associations linked to pain ratings.

### Insular spectral power shows a stable posterior pattern with hemispheric asymmetry

**Figure 2:**
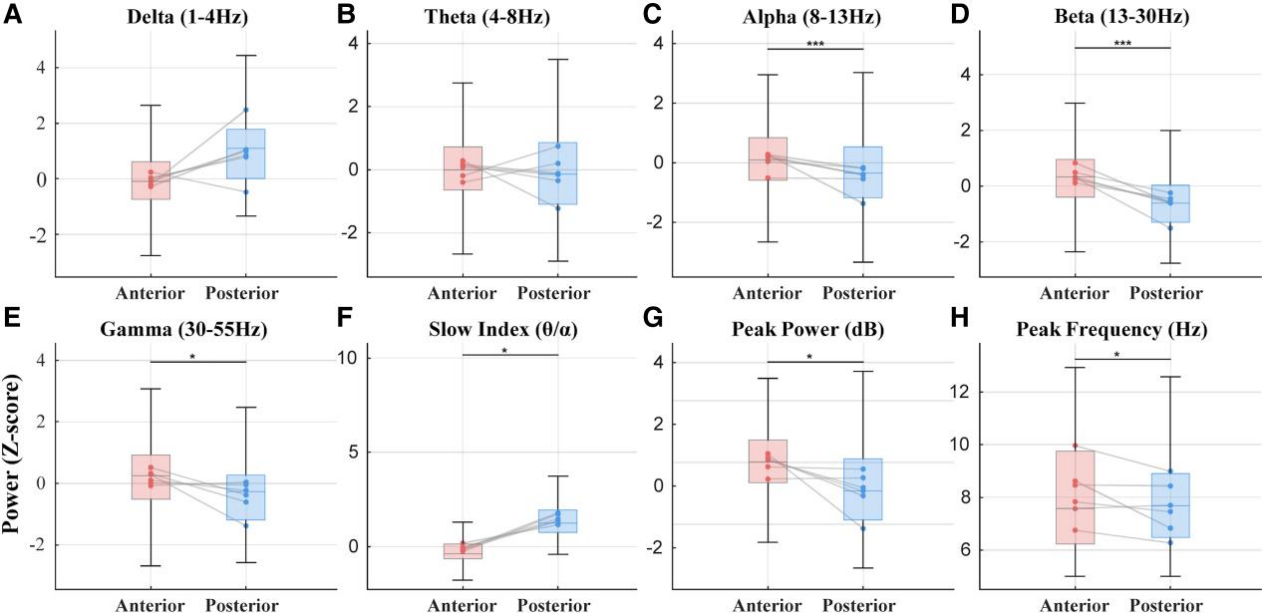
**Stable baseline spectral pattern of the human insula.** Resting-state intracranial EEG shows a reproducible anterior–posterior gradient in insular spectral organization. Power values are z-scored within subject for visualization. Paired grey lines connect participant-level anterior and posterior means, and boxplots summarize participant-level distributions (*N* = 6 participants). (**A–E**) Log-transformed spectral power in delta, theta, alpha, beta and gamma bands shows lower posterior fast-band power, most prominently in alpha (linear mixed-effects model: t = −3.14, *P* = 0.002) and beta (t = −3.59, *P* < 0.001). (**F–H**) Peak-derived spectral metrics show convergent evidence of posterior spectral slowing. Statistical inference was performed using linear mixed-effects models on channel–day observations, with random intercepts for Subject and Subject:Channel and adjustment for Day_Index_c. Full model estimates, *P* values, *q* values and partial R^2^ are reported in Supplementary Table 5.

Insular spectral power exhibited a pronounced anterior–posterior gradient after accounting for temporal drift (Fig. 2; Supplementary Table 5). This organization was driven primarily by lower alpha, beta and gamma power in posterior relative to anterior insula, rather than uniform enhancement of low-frequency power. Posterior alpha and beta power were lowest in the ipsilateral posterior quadrant (alpha: *β* ≈ −6.6 dB, *P* = 0.002; beta: *β* ≈ −7.2 dB, *P* < 0.001), with nominal additional contralateral posterior beta reduction (*β* ≈ −3.25 dB, *P* = 0.035; *q* = 0.114). These effects accounted for a meaningful proportion of variance (partial R^2^ ≈ 0.04–0.05) and were consistent across recording days.

Peak-derived spectral metrics provided convergent, though weaker, support for a posterior shift towards slower spectral composition. Posterior regions showed lower PDF and higher Slow Index values; however, these effects were nominal after correction (contralateral posterior PDF: *P* = 0.034, *q* = 0.122; Slow Index: *P* = 0.041, *q* = 0.122; Supplementary Table 5).

**Figure 3:**
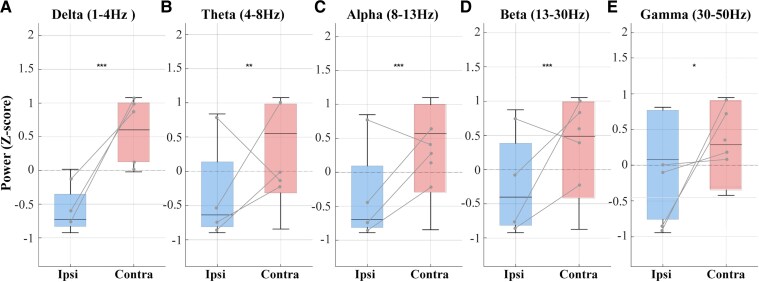
**Posterior insula spectral power is lateralized relative to the chronic pain side.** Within posterior insula, spectral power is higher in the hemisphere contralateral to the chronic pain side across multiple frequency bands. The cohort comprised *N* = 6 participants; analyses used available posterior recordings. (**A–E**) Power values are z-scored within subject for visualization. Individual data points represent available participant-level posterior regional means; grey lines connect participants with bilateral posterior data. Contralateral dominance was significant in delta (linear mixed-effects model: t = 3.52, *P* < 0.001), theta (t = 3.10, *P* = 0.003), alpha (t = 3.95, *P* < 0.001) and beta (t = 3.53, *P* < 0.001) bands, with a smaller but significant effect in gamma (t = 2.45, *P* = 0.016). Statistical inference was performed using linear mixed-effects models on channel–day observations, with Laterality and Day_Index_c as fixed effects and random intercepts for Subject and Subject:Channel. Full model estimates, *P* values, *q* values and partial R^2^ are reported in Supplementary Table 6.

Beyond the dominant anterior–posterior gradient, insular spectral power exhibited a robust lateralization relative to the side of chronic pain that was anatomically restricted to the posterior insula (Fig. 3). In posterior insula, the hemisphere contralateral to the chronic pain side showed higher spectral power than the ipsilateral hemisphere across multiple frequency bands, most prominently delta (*β* ≈ +8.5 dB, *q* = 0.002), theta (*β* ≈ +7.4 dB, *q* = 0.007), alpha (*β* ≈ +7.2 dB, *q* = 0.002) and beta (*β* ≈ +5.4 dB, *q* = 0.002), with a smaller but significant gamma effect (*β* ≈ +3.7 dB, *q* = 0.032). Pooled analyses across the entire insula did not show significant hemispheric asymmetry (Supplementary Table 6). Together, these findings indicate that laterality in chronic pain is not a whole-insula shift, but a posteriorly localized spectral imbalance.

### Pain intensity and unpleasantness are associated with frequency-specific variation in posterior alpha and beta power

**Figure 4:**
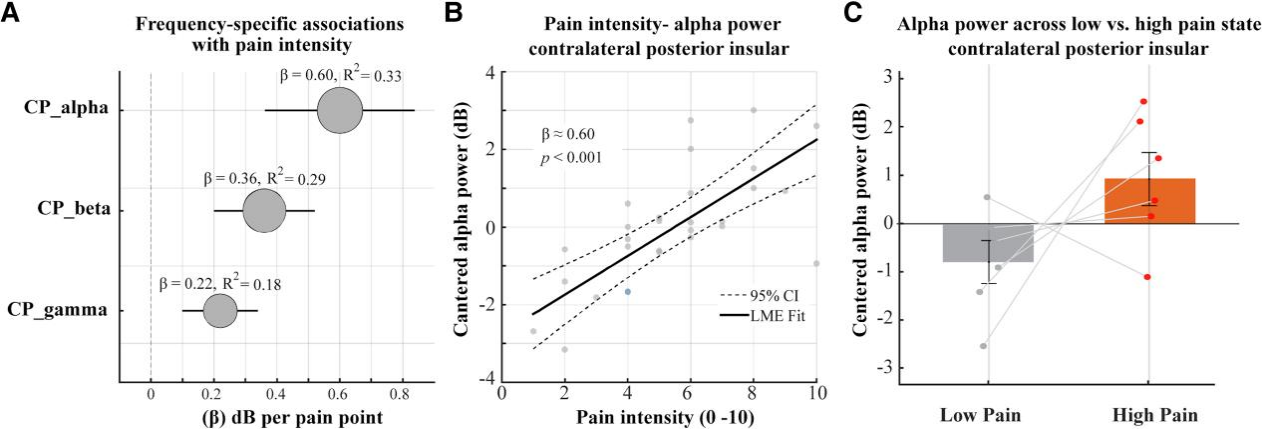
**Day-to-day association between contralateral posterior insular power and pain intensity.** The cohort comprised *N* = 6 participants; all panels use available contralateral posterior recordings with corresponding pain intensity ratings. **(A)** Mixed-effects model estimates of pain intensity associations across frequency bands in contralateral posterior insula, displaying effect sizes (β), 95% confidence intervals and partial R^2^ values derived from Supplementary Table 7. Alpha power shows the strongest association with daily pain intensity (linear mixed-effects model: t = 5.00, *P* < 0.001), with smaller but consistent effects in beta and gamma bands. **(B)** Continuous relationship between daily pain intensity and contralateral posterior alpha power. Points represent participant-day means, with alpha power centred within participant. The solid line indicates the mixed-effects model fit; dashed lines indicate 95% confidence intervals. **(C)** Contralateral posterior alpha power during low versus high pain states, defined by a within-subject median split of daily pain ratings. Bars show mean ± SEM of participant-centred alpha power (dB). Individual data points represent participant-level means for each pain state, with paired points from the same participant connected by grey lines. This panel is illustrative; statistical inference is derived from the mixed-effects models shown in **A, B.** Full statistics are reported in Supplementary Table 7.

Each one-point increase in pain intensity was associated with an approximate +0.6 dB increase in contralateral posterior alpha power (*β* = +0.60 dB, 95% CI +0.36 to +0.84, *q* < 0.001; Supplementary Table 7), accounting for a substantial proportion of within-subject variance (partial R^2^ ≈ 0.33). Pain intensity was also positively associated with contralateral posterior beta (*β* = +0.36 dB, *q* < 0.001) and gamma power (*β* = +0.22 dB, *q* = 0.014), but alpha showed the largest effect size and clearest spatial selectivity (Fig. 4).

**Figure 5:**
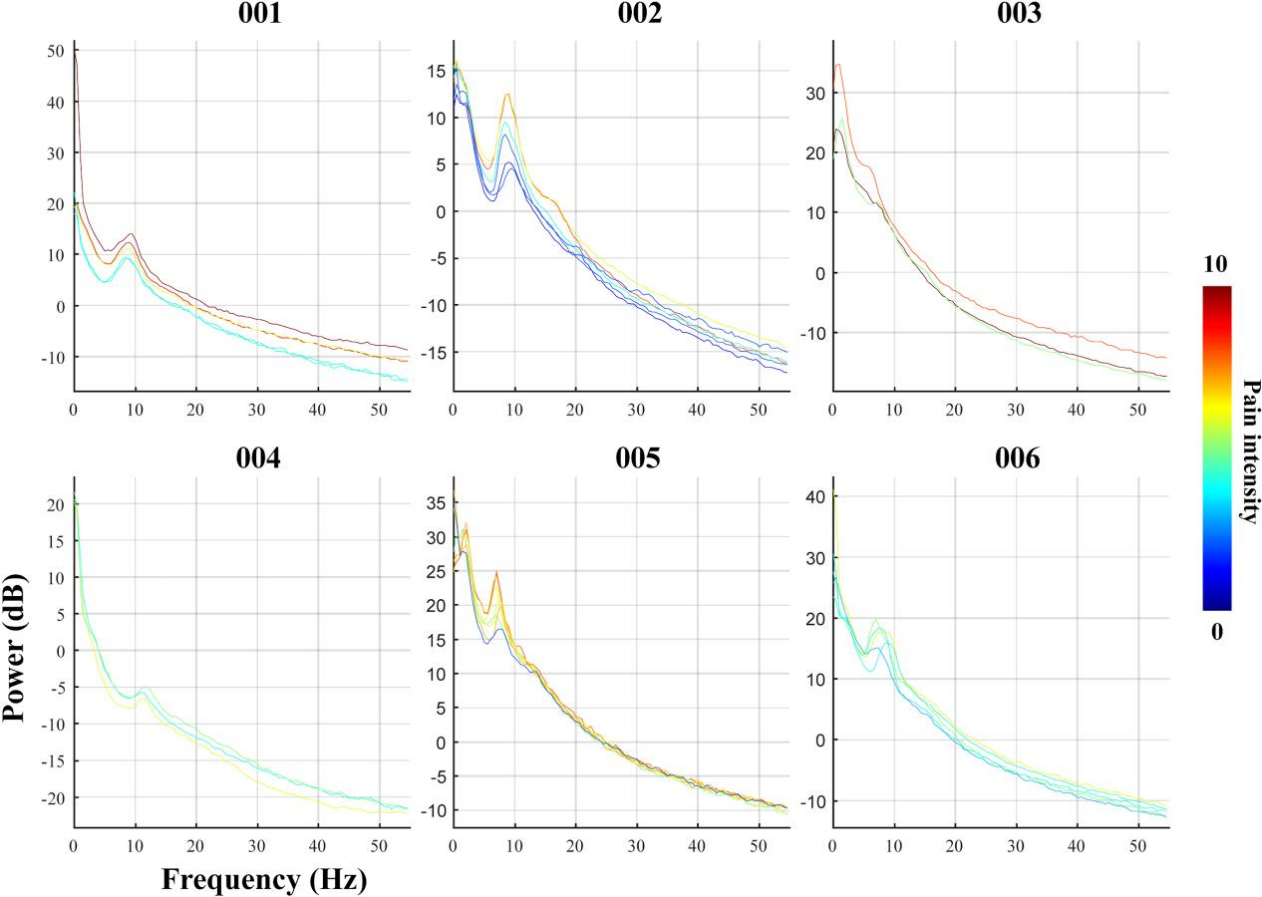
**Individual power spectral density profiles stratified by daily pain intensity.** Representative channel–day power spectral density (PSD) profiles from contralateral posterior insula illustrate increased alpha-band amplitude on higher-pain days (red) relative to lower-pain days (blue). Each trace corresponds to a single day of resting-state recording. All participants contributed both higher- and lower-pain days during monitoring. These examples illustrate the day-to-day alpha–pain associations observed in the aggregated mixed-effects analyses.

These associations persisted after controlling for temporal drift. Figure 5 illustrates contralateral posterior power spectra demonstrating increased alpha-band amplitude on higher-pain days.

**Figure 6:**
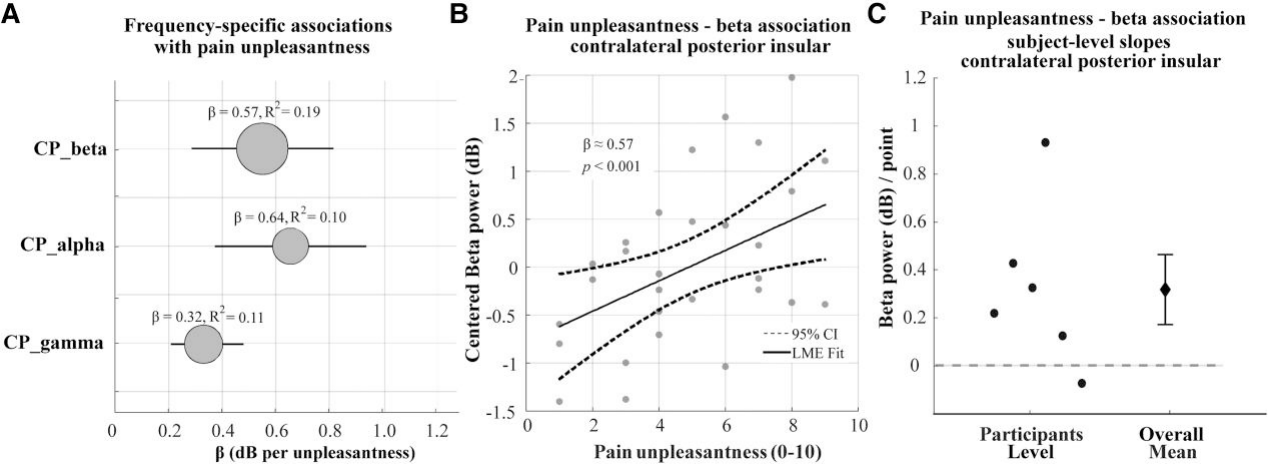
**Day-to-day association between contralateral posterior insular power and pain unpleasantness.** The cohort comprised *N* = 6 participants; all panels use available contralateral posterior recordings with corresponding daily pain–unpleasantness ratings. **(A)** Mixed-effects model estimates of pain unpleasantness associations across frequency bands in contralateral posterior insula. Points indicate effect sizes (β); horizontal bars denote 95% confidence intervals; marker size reflects partial R^2^ values reported in Supplementary Table 8. Beta-band power shows the strongest association with unpleasantness (linear mixed-effects model: t = 3.56, *P* < 0.001), whereas alpha and gamma effects are nominal and do not survive FDR correction. **(B)** Continuous relationship between daily pain unpleasantness and contralateral posterior beta power. Points represent participant-day means, with beta power centred within participant. The solid line indicates the mixed-effects model fit; dashed lines indicate 95% confidence intervals. **(C)** Participant-level slopes showing the within-participant association between daily pain unpleasantness and contralateral posterior beta power. Each data point represents a slope estimated from the available channel–day observations for one participant. The black diamond and error bar indicate mean ± SEM. This panel is illustrative; statistical inference is based on the mixed-effects models reported in Supplementary Table 8.

Pain unpleasantness exhibited a spectrally distinct pattern of day-to-day pain-related association (Fig. 6; Supplementary Table 8). The strongest effect was observed for contralateral posterior beta power (*β* = +0.57 dB, 95% CI +0.26 to +0.88, *q* = 0.027; partial R^2^ ≈ 0.19), indicating increased beta power on higher-unpleasantness days. Additional associations in contralateral posterior alpha and gamma and in ipsilateral anterior delta and gamma were nominal and did not survive FDR correction (Supplementary Table 8). Overall, unpleasantness-related associations were less spatially focal than the intensity-related alpha effect, with one robust posterior beta association alongside distributed nominal effects. Furthermore, sensitivity analyses decomposing the spectral signals into aperiodic and residual periodic components confirmed that these frequency-specific associations with pain intensity and unpleasantness remained robust. This indicates that the observed dynamics in alpha and beta power represent oscillatory variations rather than artefacts of broadband shifts in spectral slope.

Peak-derived spectral metrics showed limited sensitivity to day-level pain-related associations compared with band-limited power. Peak dominant frequency increased modestly with pain intensity in contralateral posterior insula (*β* = +0.12 Hz, *P* = 0.008; *q* = 0.024; Supplementary Table 9), indicating slight spectral shift towards higher frequencies on higher-pain days. Other peak metrics did not show robust day-to-day associations, and peak–feature variance was dominated by stable baseline patterns (Supplementary Tables 5 and 9).

### Insular connectivity shows a stable posterior–anterior organization

Independent of laterality and pain state, insular connectivity exhibited a frequency-specific posterior–anterior connectivity gradient. Gamma-band connectivity was stronger in posterior than anterior insula, most prominently for coherence (*β* ≈ +0.06, *P* = 0.002; *q* = 0.008) and with a parallel nominal effect for PLV (*β* ≈ +0.61, *P* = 0.014; *q* = 0.069; Supplementary Table 11). No consistent anterior–posterior gradients were observed for delta–beta coupling, indicating that the connectivity gradient was selective to high-frequency functional coupling.

Connectivity also exhibited frequency-specific hemispheric asymmetry relative to the chronic pain side. Spectral power showed broad posterior contralateral dominance, whereas connectivity asymmetry was confined to gamma-band networks. In posterior insula, gamma-band connectivity showed robust ipsilateral dominance for both PLV (*β* ≈ −2.00, *P* = 0.002; *q* = 0.018) and coherence (*β* ≈ −0.23, *P* < 0.001; *q* = 0.010; Supplementary Table 12). Long-range anterior–posterior insular connections showed a similar ipsilateral gamma preference for PLV (*β* ≈ −0.51, *P* < 0.001; *q* = 0.014), with no robust lateralization in lower frequency bands (Supplementary Table 12).

### Insular connectivity remains largely stable across pain states

Connectivity tracked within-subject fluctuations in pain ratings, but effect sizes were substantially smaller than those observed for spectral power. Alpha-band PLV increased with both pain intensity (*β* ≈ +0.019, *q* < 0.001) and unpleasantness (*β* ≈ +0.047, *q* = 0.002; Supplementary Table 13), consistent with a generalized alpha synchrony marker of pain level. Gamma-band PLV increased with pain intensity (*β* ≈ +0.080, *q* = 0.002) but not unpleasantness. Coherence effects were more restricted, with pain intensity positively predicting alpha coherence (*β* ≈ +0.003, *q* < 0.001), while unpleasantness showed only nominal associations in theta coherence (Supplementary Table 13). In combined models including both pain intensity and unpleasantness, few connectivity effects survived correction and effect sizes were uniformly small (Supplementary Table 14). Statistically significant effects were confined to within-subregion connections, with no evidence for robust day-to-day pain-related changes in long-range anterior–posterior insular connectivity.

## Discussion

This study provides multiday bilateral intracranial observations that resting-state insular physiology in chronic neuropathic pain is characterized by two key features: a stable within-cohort baseline spectral pattern and frequency-specific day-to-day variation associated with subjective pain ratings. Insular spectral power showed a reproducible anterior–posterior gradient, with lower posterior fast-band power and relative spectral slowing. Daily pain fluctuations were associated with selective changes in local oscillatory power rather than large-scale shifts in connectivity. Importantly, all associations were estimated with explicit control for longitudinal temporal drift, reducing the likelihood that observed pain–physiology relationships were driven by recording-related settling or inpatient effects. These findings suggest that day-to-day pain fluctuations are expressed on a stable insular spectral background, whereas intra-insular connectivity and peak-derived metrics primarily reflect enduring baseline features.

### A stable posterior spectral pattern characterizes the studied cohort

At baseline, the posterior insula showed a slow-biased spectral pattern characterized by reduced alpha, beta and gamma power compared with the anterior insula, rather than a simple increase in low-frequency activity. This anterior–posterior gradient was reproducible across recording days and remained independent of day-to-day changes in reported pain, supporting the presence of a stable baseline spectral pattern rather than a transient state effect. This pattern can be considered alongside thalamocortical dysrhythmia models of chronic pain,^11,12^ but the present data do not directly address thalamic mechanisms and indicate that the dominant feature within the insula is lower fast-band power rather than uniform low-frequency enhancement. Peak-derived metrics showed similar directional changes but were less robust, indicating that band-limited spectral amplitude provides the most sensitive measure of this persistent alteration. Baseline spectral power was also lateralized relative to the chronic pain side, with contralateral dominance confined to the posterior insula. This spatially restricted asymmetry is consistent with the lateralized organization of nociceptive pathways and supports the view that these spectral abnormalities are linked to pain-related afferent processing rather than representing a global hemispheric shift,^1,2,5^ but the absence of pain-free or disease-control intracranial recordings precludes conclusions about disease specificity or causal pain mechanisms. Importantly, this posterior spectral pattern remained stable across daily pain fluctuations, providing the physiological background upon which dynamic pain-related variation occurred.

### Day-to-day changes in insular activity across pain dimensions

Against this stable spectral organization, daily pain fluctuations were associated with partially distinct frequency-specific patterns. Pain intensity was most strongly related to contralateral posterior alpha power, whereas pain unpleasantness was associated with contralateral posterior beta power. These associations were spatially localized to the posterior insula and remained significant after controlling for longitudinal temporal drift. Since pain intensity and unpleasantness were correlated and analysed primarily in separate models, these findings should be interpreted as differential associations rather than evidence for fully dissociable encoding.

These findings refine the conventional anterior–posterior distinction often invoked to separate sensory and affective pain processing. Although the anterior insula is frequently emphasized in affective pain, accumulating evidence indicates that the posterior insula also participates in broader salience and affect-related encoding in chronic pain.^5,16,17^ The present results extend this framework by demonstrating frequency-specific differentiation within a shared posterior territory. In practical terms, different oscillatory frequency bands may relate preferentially to different aspects of the pain experience within the same cortical region.

One possible interpretation is that posterior beta activity reflects feedback influences from affective or salience-related networks onto interoceptive representations of pain, consistent with models in which beta-band activity supports top–down signaling.^18^ Although directed connectivity was not assessed, the frequency-specific separation observed here provides a physiologically coherent framework for understanding how the posterior insula may relate differently to pain intensity and unpleasantness within the same posterior region.

### Interpretation of alpha-band findings

At first glance, the findings appear paradoxical: posterior insula shows lower alpha power at baseline, yet higher alpha power on days with greater pain intensity. One speculative but physiologically plausible explanation is that day-to-day increases in alpha power reflect inhibitory regulation rather than a reversal of the underlying baseline pattern. Alpha activity has been linked to cortical inhibition and control of excitability,^19^ including modulation of pain processing.^20,21^ In this context, persistently lower baseline alpha power may reflect a chronically disinhibited state, whereas transient alpha increases on higher-pain days may represent an adaptive attempt to regulate incoming nociceptive input or stabilize local network activity. Alternative explanations, including changes in arousal or attention, cannot be excluded and require further investigation. Importantly, the alpha–pain relationship remained significant after separation of aperiodic and residual periodic spectral components and after additional adjustment for aperiodic slope, indicating that this effect cannot be explained solely by broadband spectral tilt.

### Peak-derived metrics and connectivity mainly reflect baseline organization

Peak-based measures of spectral slowing were directionally consistent with the baseline pattern but showed relatively weak associations with day-to-day pain variation. This suggests that peak-derived metrics primarily reflect stable underlying network properties, whereas band-limited power is more sensitive to short-term fluctuations in pain. Intra-insular connectivity also demonstrated a stable baseline structure with comparatively modest day-to-day variation. Baseline coupling was most evident in fast-frequency bands, particularly posterior gamma connectivity showing ipsilateral dominance, consistent with previously described patterns in chronic pain.^11,12^ Although alpha and gamma synchrony showed statistically detectable but modest associations with pain ratings, effect sizes were small relative to spectral power and limited to local connections. There was no evidence of large-scale anterior–posterior reorganization across pain states, even in more conservative combined models. These findings suggest that day-to-day fluctuations in chronic pain are expressed primarily through modulation of local oscillatory power, while connectivity and peak-derived metrics reflect relatively stable baseline organization.

### A conceptual model of posterior insular spectral associations in chronic neuropathic pain

The present findings support a model in which chronic neuropathic pain in this cohort is associated with stable insular spectral patterns, with the posterior insula emerging as a key site of frequency-specific pain-related associations. Baseline posterior spectral slowing and lower fast-band power define a persistent within-cohort physiological pattern. Superimposed on this stable background, day-to-day fluctuations in pain intensity were most strongly associated with alpha-band variation, whereas fluctuations in pain unpleasantness were primarily associated with beta-band variation within the same posterior region. In this model, the posterior insula does not separate sensory and affective pain dimensions by anatomical location alone. Instead, different frequency bands within the same cortical region show partially distinct associations with different aspects of pain experience. Posterior spectral power therefore functions as a candidate dynamic indicator of pain-related brain state, while intra-insular connectivity and peak-derived measures reflect comparatively stable underlying network structure.

### Methodological considerations

The rarity of bilateral, multiday insular implantation necessarily limits cohort size. Although only six participants were included, dense within-subject sampling across 34 recording days enabled hierarchical modelling of day-level associations nested within individuals. This design minimizes reliance on between-subject comparisons but limits generalizability across pain aetiologies and phenotypes. Replication in larger cohorts will be required to confirm robustness and clinical applicability.

Given the small cohort size and the number of anatomical regions, frequency bands, connectivity metrics and behavioural outcomes examined, the present findings should be considered exploratory and hypothesis-generating. The observed spectral associations identify candidate physiological signals that require replication in larger and independent cohorts before disease specificity, generalizability or clinical biomarker utility can be established.

The absence of a healthy comparator group reflects the ethical constraints of invasive recordings. Accordingly, inference relied on internal anatomical contrasts and within-subject longitudinal variation. While this approach strengthens mechanistic interpretation, it does not replace population-level comparisons.

### Clinical and mechanistic implications

Posterior insular alpha and beta oscillations emerge as candidate physiological signals differentially associated with pain intensity and unpleasantness, respectively. These findings support a framework in which the posterior insula functions primarily as a sensing region rather than an optimal stimulation target. Given the sensory and visceral risks associated with posterior insular stimulation,^2^ closed-loop strategies may use posterior spectral features for real-time state estimation while delivering modulation to more clinically tolerable network targets. Prospective studies will be required to establish causal relationships and therapeutic utility.

## Conclusion

Chronic neuropathic pain in this cohort was associated with stable insular spectral patterns upon which frequency-specific day-to-day variation related to pain ratings. Band-limited spectral power provides the most sensitive representation of dynamic pain-related variation, whereas connectivity and peak-derived metrics reflect more stable baseline features. These exploratory findings identify posterior insular alpha–beta activity as a candidate physiological sensing signal with potential relevance for adaptive neuromodulation, requiring validation in larger independent cohorts before biomarker or therapeutic applications can be established.

## Supplementary Material

fcag299_Supplementary_Data

## Data Availability

Data are available from the corresponding author upon reasonable request. The dataset is currently being prepared for public release via the DABI repository. Code used for preprocessing, spectral analysis, connectivity analysis, aperiodic residual analysis and statistical modelling is available at https://github.com/UVA-LIU/UVA-LIU-uva-insular-iEEG-neuropathic-pain-analysis.
