# Supplementary material for: Stable insular spectral patterns underlie dynamic pain encoding in chronic neuropathic pain
Source: Brain Commun. 2026 Aug 3;8(4):fcag299. doi: 10.1093/braincomms/fcag299 (PMC13457931; doi:10.1093/braincomms/fcag299)
Supplement: Supplementary file 1 [file fcag299_supplementary_data.pdf]

SUPPLEMENTARY STATISTICAL METHODS:

Mixed-Effects Models for Stable Baseline Spectral Organization

Stable baseline spectral organization of the insula was assessed using linear mixed-effects models applied to resting-state intracranial EEG (iEEG) spectral features. These models were designed to quantify persistent anatomical patterns of insular oscillatory activity that are conserved across recording days, independent of day-to-day pain-related variation in pain intensity or unpleasantness.

Data Aggregation and Unit of Analysis

To strictly control for pseudo-replication and unequal epoch counts, all analyses were conducted at the channel–day aggregated level. Prior to aggregation, artifact rejection was performed through comprehensive visual inspection of the continuous raw recordings. Given the high signal-to-noise ratio of intracranial recordings, exclusion criteria focused on non-physiological transients (e.g., amplifier saturation, railing), unstable contact artefacts, or interictal epileptiform discharges. For each channel on each recording day, spectral metrics were averaged across all retained artefact-free epochs. Only channel–day observations with sufficient data quality were retained for analysis.

Model Specification

For each canonical frequency band (delta, theta, alpha, beta, gamma) and each peak-derived spectral metric, the following linear mixed-effects model was fit:

Spectral_Metric ~ 1 + Quad_Region + Day_Index_c + (1 | Subject) + (1 | Subject:Channel)

Fixed Effects

- Quad_Region: A categorical variable encoding the four insular quadrants (ipsilateral-anterior, ipsilateral-posterior, contralateral-anterior, contralateral-posterior).
- Day_Index_c: A continuous, subject-mean centred covariate representing recording day. This explicitly models and removes slow temporal drift specific to each participant's inpatient monitoring period.

Random Effects

- (1 | Subject): Random intercepts accounted for between-subject differences in baseline spectral power.
- (1 | Subject:Channel): Nested random intercepts accounted for stable, electrode-specific variability within each subject (e.g., impedance differences or precise anatomical placement).

Reference Coding and Interpretation

Models used *ipsilateral anterior insula* as the reference category. Consequently:

- Positive β coefficients indicate higher spectral values relative to the anterior–ipsilateral baseline (e.g., a positive β for contralateral posterior delta reflects posterior slow-wave enhancement).
- Negative β coefficients indicate lower spectral values relative to the anterior–ipsilateral baseline.

Statistical Inference

Significance of fixed effects was assessed using t-statistics derived from the fitted mixed-effects models. P values were corrected for multiple comparisons using Benjamini-Hochberg false-discovery-rate (FDR) procedures applied across all band-by-quadrant combinations within each spectral family. Effect sizes are reported as partial R^2, computed from t-statistics as $t^2/(t^2 + df)$, representing the proportion of residual variance uniquely explained by each fixed effect.

Mixed-Effects Models for Day-to-Day Pain-Related Associations with Spectral Power

Day-to-day pain-related associations between insular electrophysiology and chronic pain were assessed using subject-centered linear mixed-effects models. These models were designed to quantify within-subject covariation between resting-state insular activity and subjective pain ratings after accounting for stable baseline anatomical organization and slow temporal drift.

Model Specification

To allow the relationship between pain and neural activity to vary freely across anatomical subregions, separate mixed-effects models were fit for each insular quadrant. For each frequency band within a quadrant, the following model was fit to the channel–day aggregated data:

Neural_Metric ~ 1 + DayIndex_c + Pain_Rating_c + (1 | Subject) + (1 | Subject:Channel)

Fixed Effects

- **Day_Index_c**: Subject-mean centred covariate controlling for longitudinal drift.
- **Pain_Rating_c (Intensity or Unpleasantness):** Continuous, subject-mean centred predictor isolating within-subject day-to-day fluctuations relative to each participant's own mean pain level.

This centering strategy ensures that pain-related slopes reflect within-subject associations rather than between-subject differences.

Separation of Pain Dimensions

To avoid multicollinearity between pain intensity and unpleasantness ratings, these dimensions were evaluated in separate parallel model families, enabling independent assessment of sensory and affective pain signatures.

Random Effects

- **(1 | Subject) + (1 | Subject:Channel)**: Identical to the trait models, these intercepts account for baseline differences across subjects and individual electrodes.

Interpretation

Within this framework, the fixed effect of Pain_Rating_c represents the day-level association between pain ratings and neural activity (e.g., dB change per one-point increase in pain), independent of temporal drift and baseline anatomical organization.

Statistical Inference

Significance was assessed using t-statistics, with FDR correction applied within predefined families of tests (4 quadrants × 5 frequency bands). Effect sizes are reported as partial R^2.

Mixed-Effects Models for Insular Connectivity

Insular functional connectivity was assessed using linear mixed-effects models applied to phase-locking value (PLV) and amplitude-based coherence metrics computed between pairs of intracranial insular channels. Connectivity analyses were restricted to within-insula channel pairs.

Data Aggregation and Unit of Analysis

Connectivity metrics were aggregated at the pair–day level. For each channel pair on each recording day, connectivity values were averaged across all artefact-free epochs. Only pair–day observations meeting quality-control criteria were retained.

Prior to modeling, coherence values were Fisher Z-transformed and PLV estimates were logit-transformed to stabilize variance and approximate normality. Results were back-transformed for visualization where appropriate.

Baseline Connectivity Organization

To assess stable anatomical organization of insular connectivity independent of pain level, the following model was fit for each frequency band and connectivity metric:

Connectivity_Metric ~ 1 + Axis + Laterality + DayIndex_c + (1 | Subject) + (1 | Subject:Channel_Pair)

Where:

- **Axis** encoded anterior vs posterior insular pairing.
- **Laterality** encoded ipsilateral vs contralateral pairing relative to the chronic pain side.

Day-to-Day Pain-Related Connectivity Associations

Day-to-day pain-related associations of connectivity were assessed using subject-centred models:

Connectivity_Metric ~ 1 + DayIndex_c + Pain_Rating_c + (1 | Subject) + (1 | Subject:Channel_Pair)

Pain intensity and unpleasantness were evaluated in separate models to avoid multicollinearity.

Combined Models (Sensitivity Analysis)

As a conservative sensitivity analysis, combined models including both pain dimensions and explicit anatomical terms were fit:

Connectivity_Metric ~ PainIntensity_c + Unpleasantness_c + Axis × Laterality + DayIndex_c + (1 | Subject) + (1 | Subject:Channel_Pair)

These models assessed shared variance between pain dimensions and the robustness of connectivity effects under stricter specifications (Supplementary Table 14).

Reference Coding and Interpretation

For connectivity models, anterior and ipsilateral pairings served as reference categories. Positive β coefficients indicate stronger connectivity relative to these baselines, whereas negative coefficients indicate lower connectivity relative to the reference categories.

Statistical Inference

Statistical inference and FDR correction followed the same procedures as spectral power analyses, with corrections applied within predefined Metric × Band × Anatomical families. Effect sizes are reported as partial R^2, computed from t-statistics as partial $R^2 = t^2 / (t^2 + df)$.

Rationale

This modeling framework enables rigorous dissociation of stable baseline anatomical organization from day-to-day pain-related neural associations. Subject-centering of pain ratings isolates within-subject pain-related variance, while inclusion of Day_Index_c removes slow recording drift. Together, these approaches ensure that significant pain-related effects reflect day-level associations with insular physiology, whereas null effects indicate stability across fluctuations in reported pain.

Additional sensitivity analyses were performed to separate broadband aperiodic structure from residual oscillatory peak activity; these procedures are described below

As a sensitivity analysis prompted by reviewer concern regarding potential contribution of the aperiodic spectral component, we performed an additional decomposition of resting-state power spectra into aperiodic and residual periodic components. This analysis was designed to test whether the observed pain-related alpha and beta associations could be explained solely by changes in broadband spectral slope.

Spectral parameterisation procedure

For each retained channel-day spectrum, the aperiodic component was estimated by fitting a linear function in log-log space, using $\log_{10}$ power as a function of $\log_{10}$ frequency. To reduce bias from prominent oscillatory peaks, the canonical alpha (8–13 Hz) and beta (13–30 Hz) ranges were excluded from the aperiodic fit. The fitted line yielded two parameters:

- Aperiodic offset, representing the broadband intercept of the spectrum
- Aperiodic slope, representing the steepness of spectral decay across frequencies

The fitted aperiodic component was then subtracted from the original log-transformed spectrum to generate a residual spectrum, intended to isolate periodic peak structure independent of broadband slope.

Residual spectral metrics

From the residual spectrum, we extracted:

Residual alpha peak height, defined as the maximal residual amplitude within 8–13 Hz

Residual beta peak height, defined as the maximal residual amplitude within 13–30 Hz

These residual measures quantify oscillatory peak prominence above the fitted aperiodic background rather than raw band-limited power.

Statistical modeling

Residual alpha and beta peak metrics were analyzed using the same channel-day aggregated linear mixed-effects framework used in the primary spectral analyses. Models included fixed effects for pain rating and anatomical terms, with random intercepts for Subject and Channel nested within Subject.

To test whether oscillatory peak effects persisted independently of the broadband background, additional models included aperiodic slope as a covariate. This allowed direct comparison of:

1. the association between pain ratings and the residual periodic peak measure
2. the association between pain ratings and the aperiodic component itself
3. whether the periodic effect remained significant after adjustment for aperiodic slope

Interpretation

This analysis was treated as a sensitivity test rather than a replacement for the primary spectral models. Its purpose was to determine whether the primary pain-related findings, especially the contralateral posterior alpha association with pain intensity, reflected genuine oscillatory variation or were attributable mainly to broadband spectral tilt.

SUPPLEMENTARY TABLE 1.

Subject	Clinical profiles
001	A 79 year old woman with severe left lower extremity neuropathic pain following multiple lumbar and thoracolumbar spine surgeries, including decompression, fusion, laminotomy, and nerve transection. Her pain began after extensive decompression and facetectomy and worsened after a left L5 ganglionectomy. The most painful region was the left ankle and dorsal foot. She described constant burning and electrical pain with marked inability to bear weight on the left side despite extensive prior surgical and medical treatment.
002	A 69 year old woman with chronic severe left facial neuropathic pain involving the left V1 to V3 trigeminal distribution and left scalp. Her pain began approximately 10 years earlier in the left temporal region and was refractory to extensive prior interventions, including bilateral auriculotemporal nerve blocks, left supraorbital and infraorbital peripheral nerve stimulation trial, microvascular decompression, gamma knife radiosurgery, and glycerol injection. She described constant burning and stinging pain with intermittent sharp shooting exacerbations and marked tactile allodynia affecting the left face.
003	A 68 year old man with chronic left hemibody and facial central neuropathic pain following a right thalamic infarct. His most severe pain involved the left palm and upper extremity, described as constant squeezing pain that could radiate proximally to the elbow and neck with movement. He also reported severe burning and stinging pain involving the left forehead and medial canthus. Symptoms were persistent, disabling, and aggravated by activity despite multiple efforts at symptom control.
004	A 43 year old woman with right hemibody central post stroke pain following a remote left lateral medullary infarct. Several months after the stroke, she developed constant right sided burning pain, deep aching, and hypersensitivity involving the hemibody. Although her initial focal neurological deficits largely resolved, the neuropathic pain persisted and remained functionally significant despite prior medical treatment.
005	A 59 year old woman with right sided trigeminal neuropathic pain following resection of a right petroclival meningioma, complicated by postoperative trigeminal numbness, facial weakness, and hearing loss. She subsequently developed persistent painful dysesthesia in the right trigeminal distribution. She described constant severe right facial pain with marked tactile trigger sensitivity, exacerbated by weather, stress, and light facial contact. Prior treatments included trigeminal nerve blocks, ablations, botulinum toxin injections, acupuncture, and gamma knife radiosurgery, none of which provided durable relief.
006	A 70 year old man with left sided thalamic pain syndrome following a right thalamic stroke. His pain began approximately one month after the stroke and involved the left face, arm, chest, and leg, with greatest severity in the left arm, shoulder, and ribcage. He described pins and needles, burning, and aching pain that remained severe despite multimodal medical therapy, including gabapentin, amitriptyline, tramadol, and acetaminophen.

SUPPLEMENTARY TABLE 2. ELECTRODE LOCALIZATION

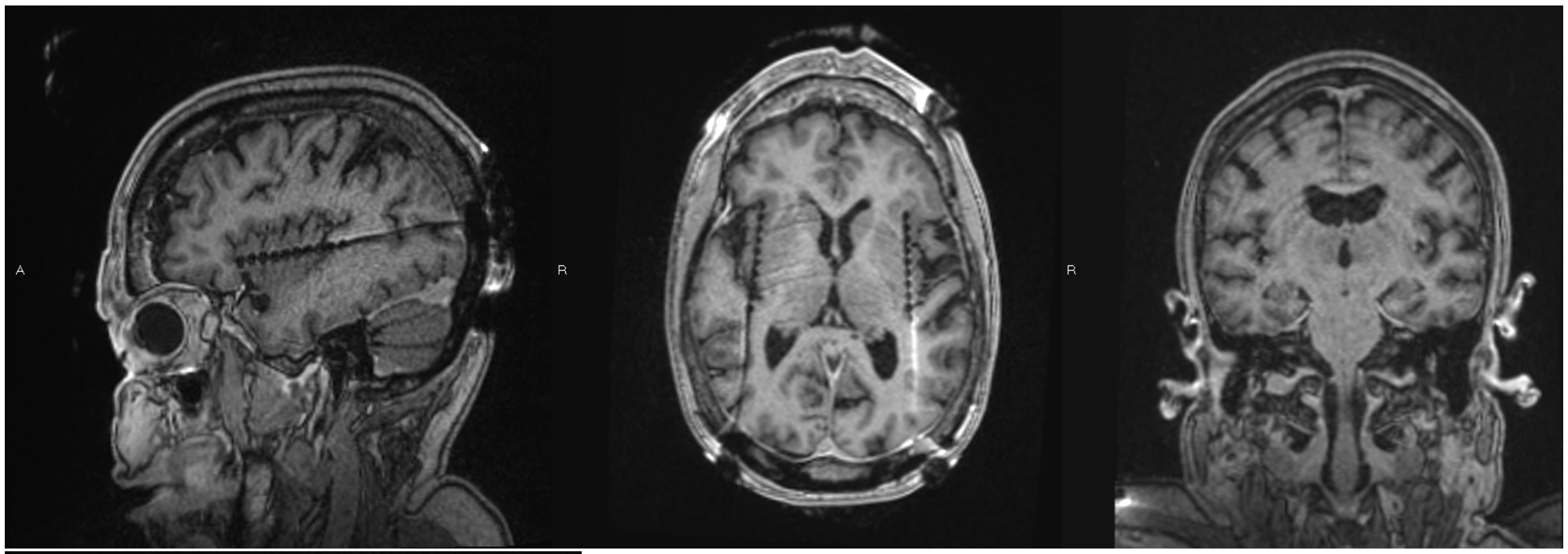

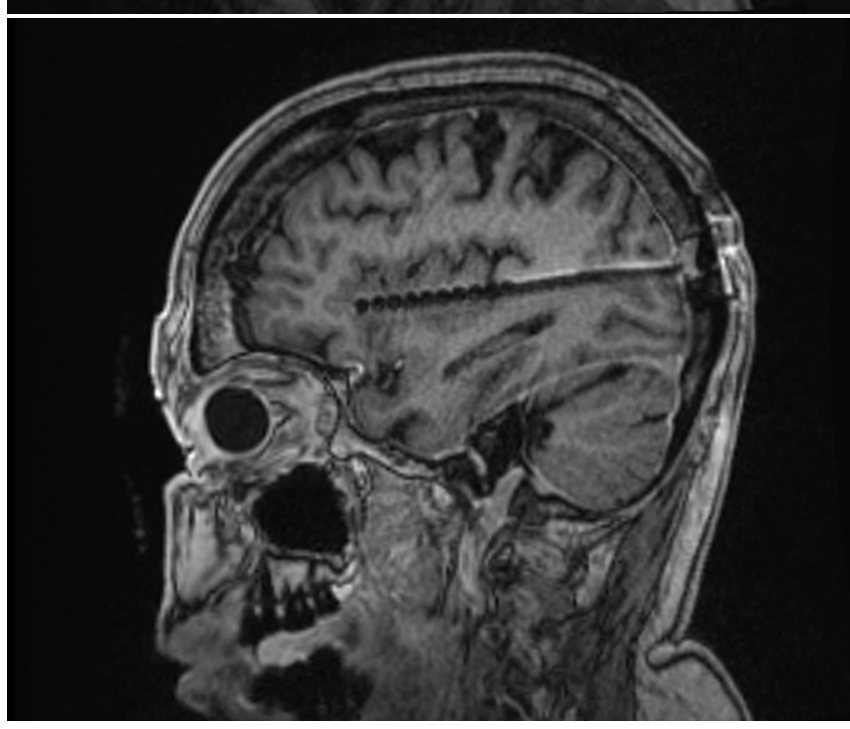

001

**Contact**	**x**	**y**	**z**	**AP**	**Contact**	**x**	**y**	**z**	**AP**
R1	32.62	21.76	-1.73	Anterior	L1	-28.59	20.23	4.27	Anterior
R2	32.65	15.99	-0.81	Anterior	L2	-29.56	14.02	4.78	Anterior
R3	32.68	10.00	-0.11	Anterior	L3	-29.54	8.67	5.20	Anterior
R4	*32.70*	*5.06*	*0.07*		L4	-29.51	2.90	5.53	Anterior
R5	32.72	-1.21	0.52	Posterior	*L5*	*-30.49*	*-2.69*	*5.48*	
R6	32.75	-6.47	1.34	Posterior	L6	-30.46	-8.31	5.67	Posterior
R7	34.77	-12.26	2.07	Posterior	L7	-31.44	-13.67	6.14	Posterior
R8	34.80	-18.30	2.78	Posterior	L8	-32.41	-19.51	6.99	Posterior
R9	*35.82*	*-24.01*	*3.46*		*L9*	*-33.39*	*-25.51*	*7.09*	
R10	*35.85*	*-29.41*	*4.81*		*L10*	*-33.36*	*-30.98*	*7.36*	

	**Central Sulcus**		
**Side**	**x**	**y**	**z**
**R**	33.71	2.23	0.91
**L**	-31.48	-3.81	7.41

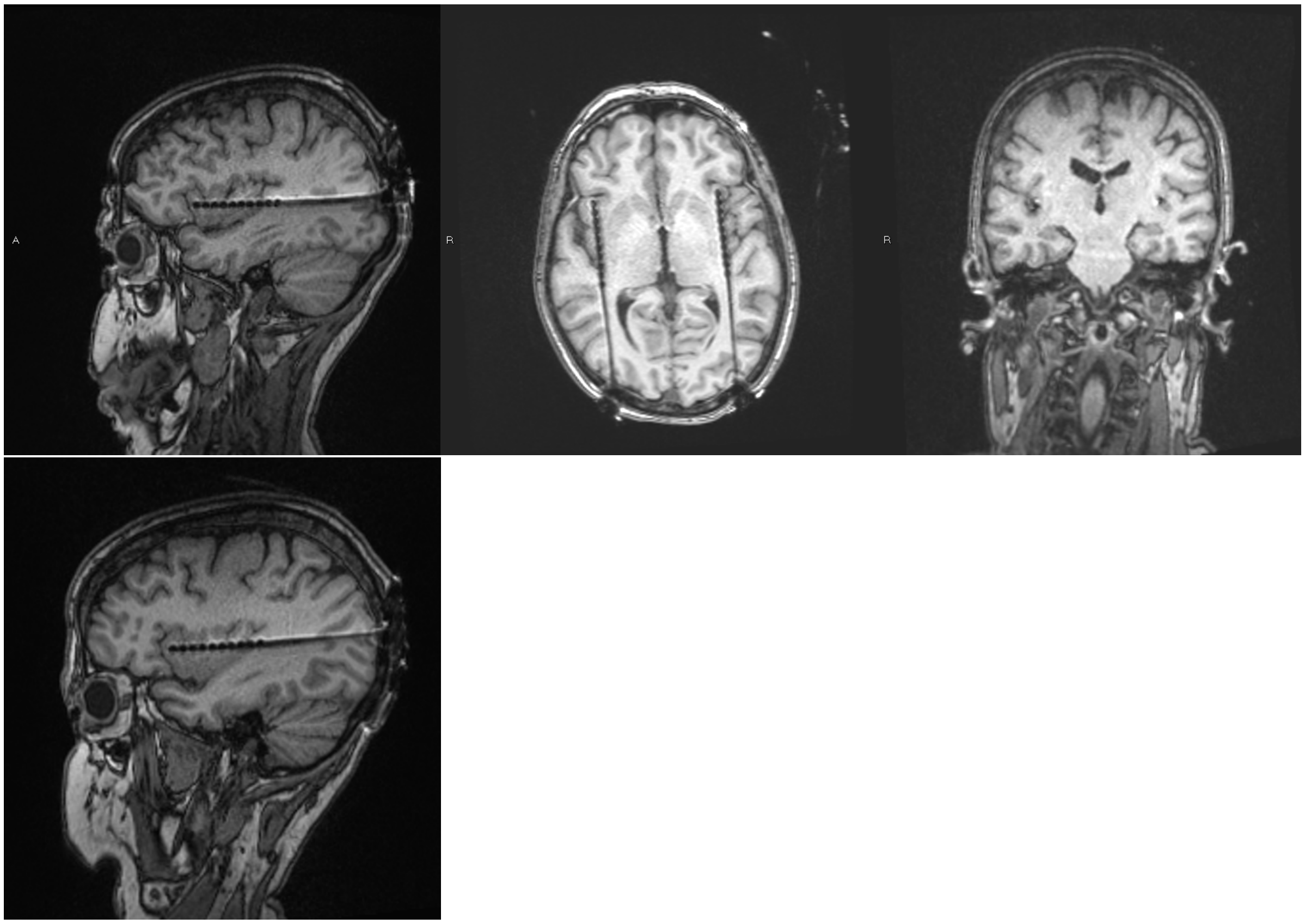

002

Contact	x	y	z	AP	Contact	x	y	z	AP
R1	36.40	16.53	-4.67	Anterior	L1	-31.13	17.49	-5.67	Anterior
R2	36.60	10.89	-4.23	Anterior	L2	-30.95	12.62	-4.77	Anterior
R3	36.77	6.33	-3.96	Anterior	L3	-31.76	7.21	-3.72	Anterior
R4	*36.93*	*1.81*	*-2.48*		L4	-31.59	2.64	-2.91	Anterior
R5	37.14	-3.88	-2.12	Posterior	L5	-32.42	-1.98	-2.00	Anterior
R6	36.33	-8.94	-0.47	Posterior	*L6*	*-32.23*	*-7.27*	*-1.41*	
R7	36.48	-13.16	0.10	Posterior	L7	-33.07	-11.62	-0.01	Posterior
R8	36.65	-17.94	0.59	Posterior	L8	-32.89	-16.57	0.52	Posterior
R9	*36.85*	*-23.35*	*1.41*		*L9*	*-33.40*	*-21.14*	*2.19*	
R10	*36.02*	*-28.20*	*2.62*		*L10*	*-34.03*	*-26.33*	*2.64*	

Central Sulcus

Side	x	y	z
**R**	36.54	1.41	-2.30
**L**	-33.99	-7.56	-0.83

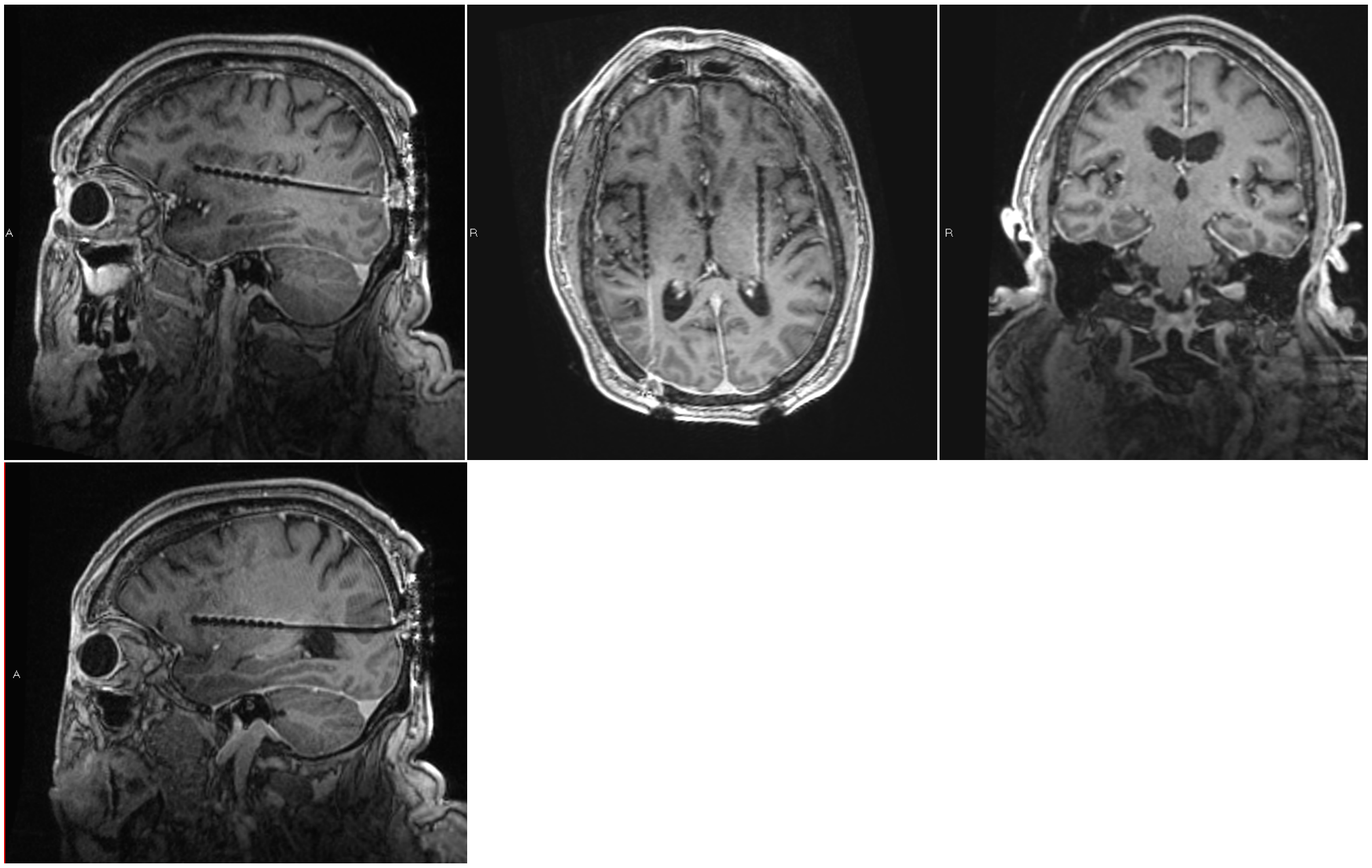

003

Contact	x	y	z	AP	Contact	x	y	z	AP
R1	32.02	20.12	-4.29	Anterior	L1	-31.43	19.01	-7.76	Anterior
R2	32.05	15.07	-3.34	Anterior	L2	-31.22	13.73	-6.29	Anterior
R3	33.13	9.54	-1.36	Anterior	L3	-30.27	8.30	-4.47	Anterior
R4	33.16	5.04	-0.55	Posterior	L4	-29.49	3.36	-2.46	Anterior
R5	33.94	-0.95	0.80	Posterior	L5	-29.01	-1.50	-0.98	Anterior
R6	33.97	-5.86	1.68	Posterior	*L6*	*-28.12*	*-6.65*	*0.65*	
R7	34.68	-10.83	2.67	Posterior	L7	-27.40	-11.85	2.39	Posterior
R8	34.79	-16.75	4.34	Posterior	L8	-26.90	-16.91	3.81	Posterior
R9	*35.22*	*-21.52*	*5.52*		L9	-26.05	-22.18	5.73	Posterior
R10	*35.51*	*-26.24*	*6.38*		*L10*	*-25.28*	*-26.38*	*7.22*	

Central Sulcus

Side	x	y	z
**R**	33.26	7.63	-1.16
**L**	-34.69	-6.17	1.04

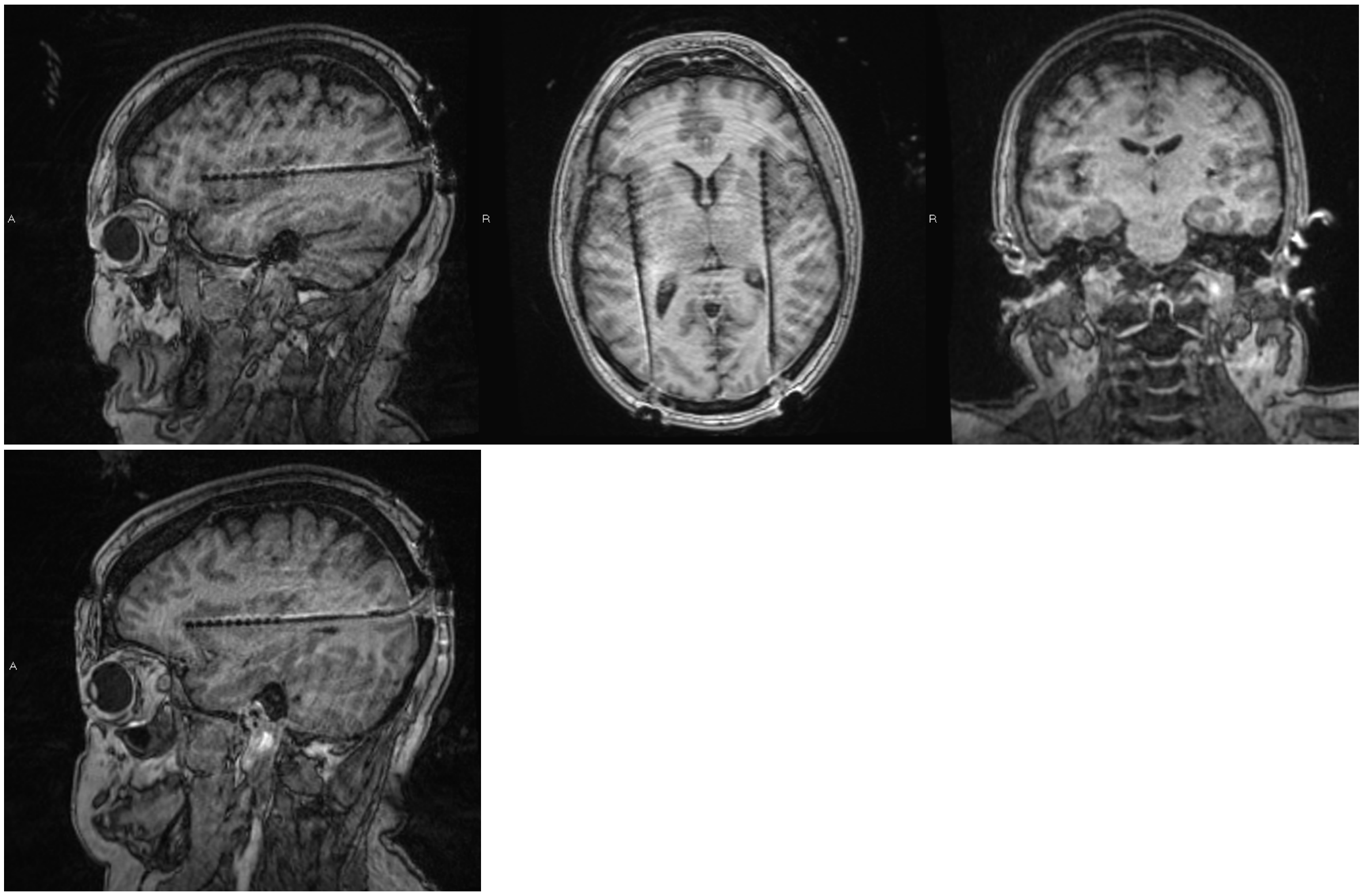

004

Contact	x	y	z	AP	Contact	x	y	z	AP
R1	39.20	18.10	-1.15	Anterior	L1	-30.18	24.16	-1.66	Anterior
R2	39.63	12.78	-0.06	Anterior	L2	-29.68	18.81	-0.95	Anterior
R3	39.77	7.99	0.37	Anterior	L3	-29.07	14.02	-0.44	Anterior
R4	39.89	3.25	1.16	Anterior	L4	-28.93	9.30	0.98	Anterior
R5	39.64	-1.94	2.51	Anterior	L5	-28.65	4.25	1.82	Anterior
R6	*39.74*	*-6.55*	*3.92*		L6	-28.43	-0.73	2.71	Anterior
R7	39.25	-11.68	4.57	Posterior	L7	-28.15	-5.90	3.49	Posterior
R8	39.23	-16.38	5.78	Posterior	L8	-27.79	-10.48	3.92	Posterior
R9	*39.26*	*-21.62*	*7.25*		L9	-27.98	-15.13	5.16	Posterior
R10	*38.88*	*-26.20*	*7.94*		*L10*	*-27.87*	*-20.85*	*5.98*	

Central Sulcus

Side	x	y	z
**R**	37.81	-8.27	3.24
**L**	-28.11	-4.75	3.98

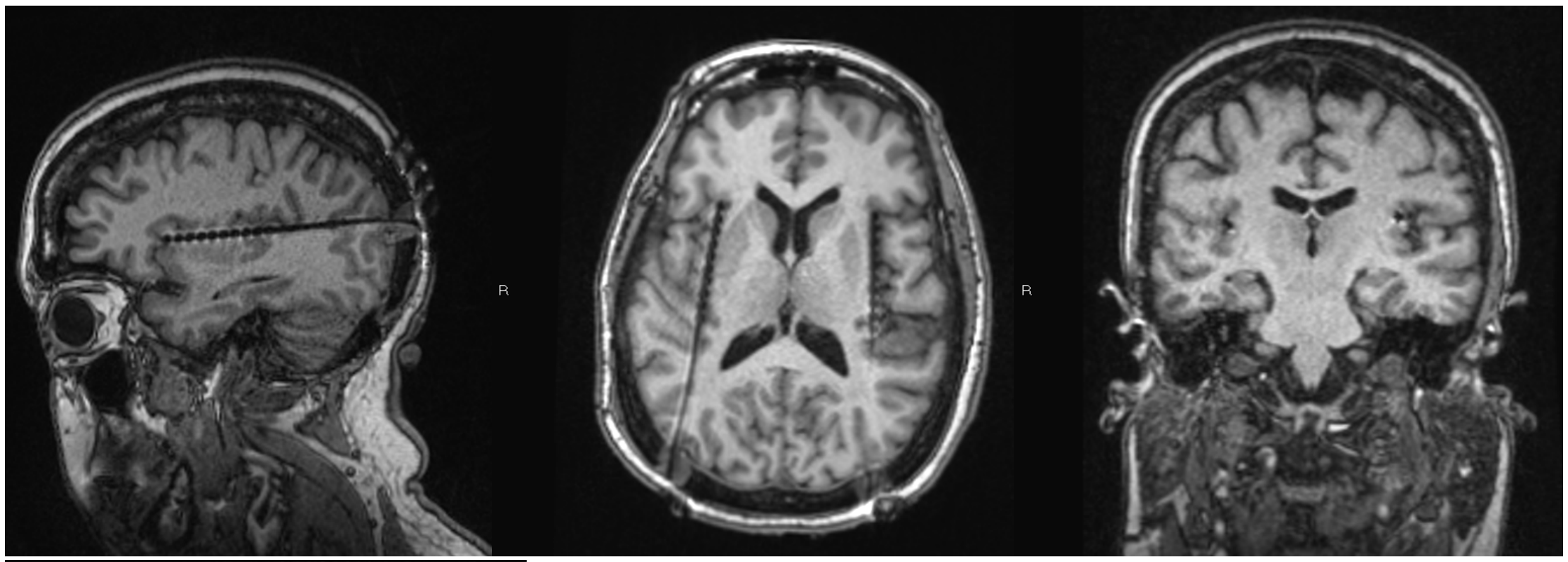

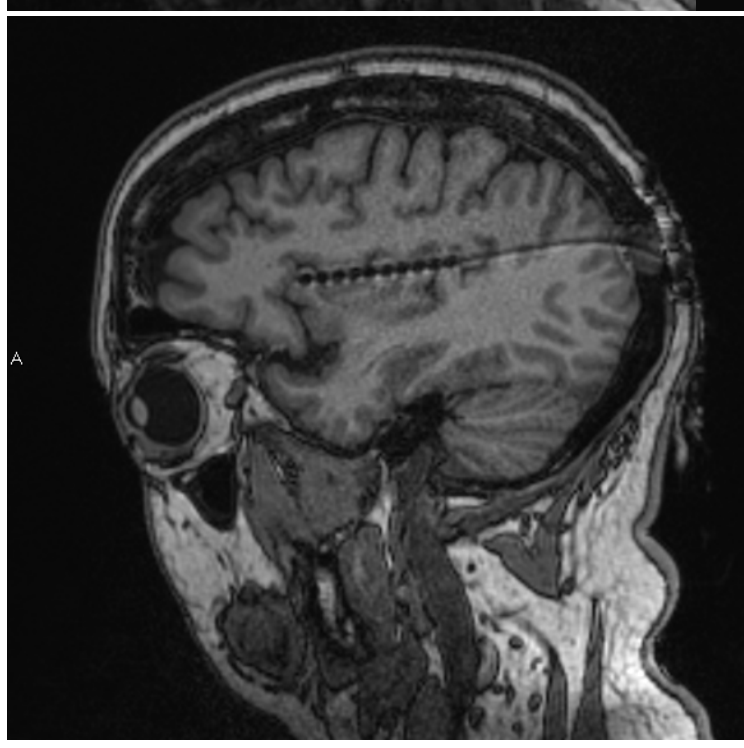

005

Contact	x	y	z	AP	Contact	x	y	z	AP
R1	29.82	18.87	3.74	Anterior	L1	-33.55	18.92	4.88	Anterior
R2	30.23	13.41	4.39	Anterior	L2	-33.67	13.83	6.11	Anterior
R3	30.93	8.79	4.81	Anterior	L3	-33.78	8.90	7.20	Anterior
R4	31.96	3.71	5.64	Anterior	L4	-33.88	4.36	8.11	Anterior
R5	32.72	-1.68	6.42	Anterior	L5	-34.22	-1.88	9.52	Anterior
R6	*33.07*	*-7.21*	*7.05*		L6	-34.31	-6.10	10.22	Anterior
R7	32.97	-11.81	8.11	Posterior	*L7*	*-34.59*	*-12.69*	*11.72*	
R8	34.07	-17.51	9.34	Posterior	L8	-34.70	-17.44	12.82	Posterior
R9	34.91	-22.72	9.61	Posterior	L9	-34.84	-23.39	13.76	Posterior
R10	*35.52*	*-28.06*	*10.98*		*L10*	*-34.96*	*-28.93*	*15.38*	

Central Sulcus

Side	x	y	z
**R**	33.11	-8.64	7.80
**L**	-34.23	-10.41	12.10

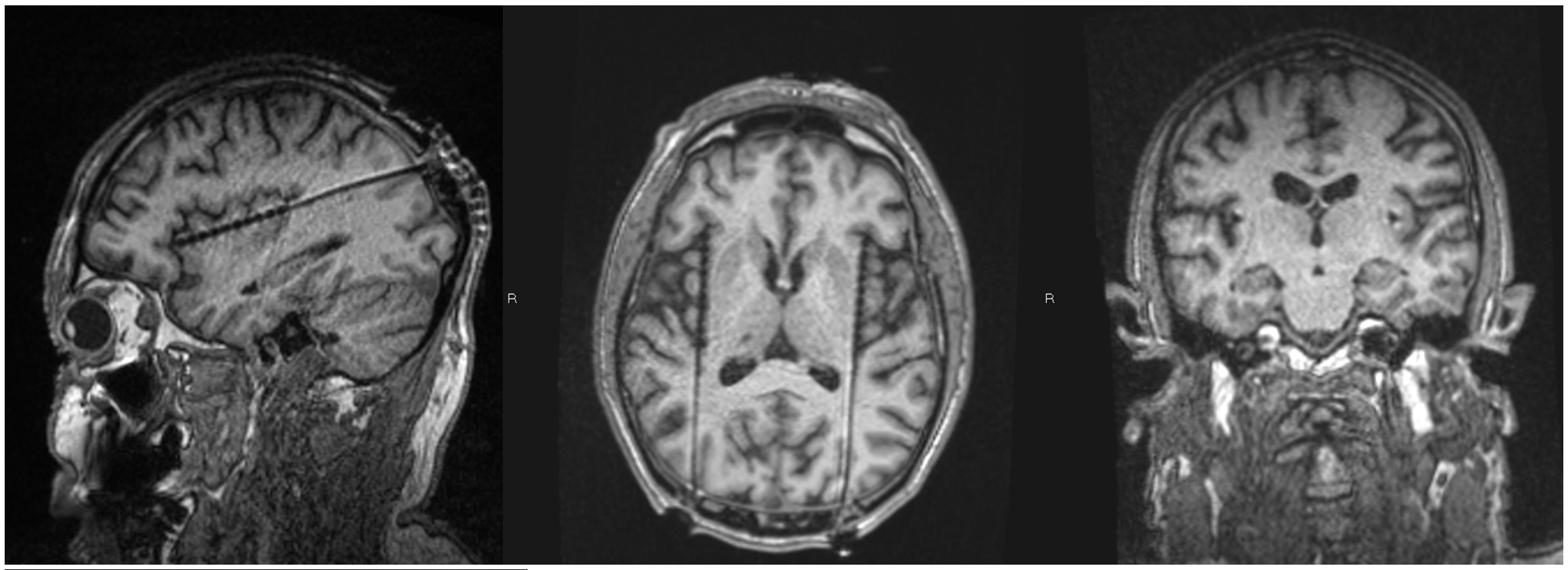

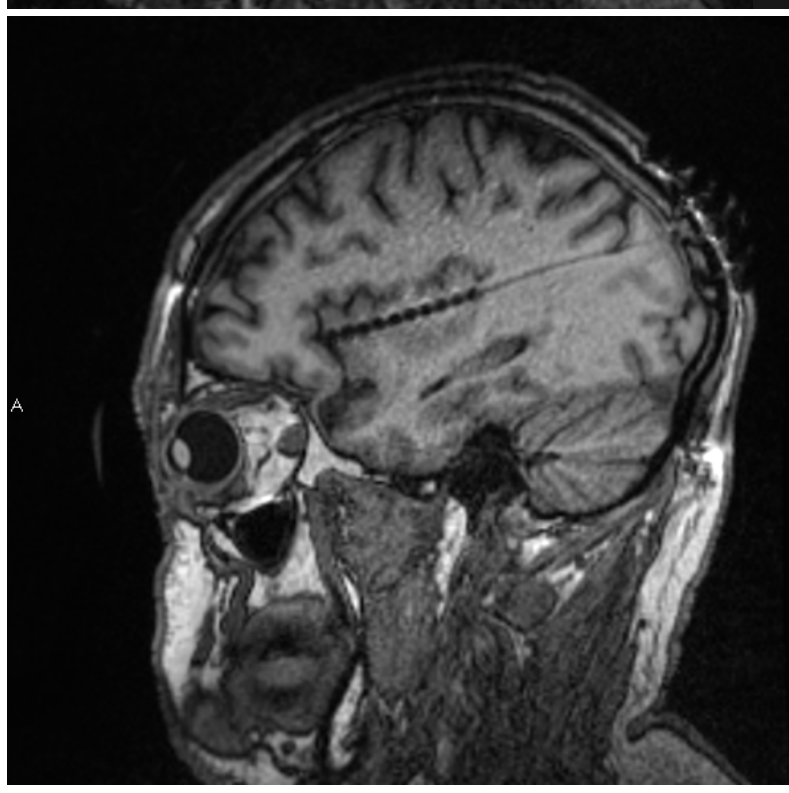

006

Contact	x	y	z	AP	Contact	x	y	z	AP
R1	34.99	19.29	-2.46	Anterior	L1	-33.04	19.72	-5.26	Anterior
R2	35.33	14.98	-1.65	Anterior	L2	-32.76	14.62	-3.95	Anterior
R3	34.55	10.12	0.36	Anterior	L3	-32.79	10.13	-2.54	Anterior
R4	35.20	5.43	2.10	Anterior	L4	-32.82	5.62	-0.78	Anterior
R5	34.85	0.27	3.79	Anterior	L5	-31.85	-0.55	1.23	Anterior
R6	34.82	-4.78	6.19	Anterior	L6	-31.88	-4.02	2.44	Anterior
R7	34.79	-9.6	7.43	Posterior	*L7*	*-31.91*	*-9.41*	*4.17*	
R8	34.77	-12.85	8.98	Posterior	L8	-31.94	-14.25	5.51	Posterior
R9	34.74	-17.87	10.00	Posterior	L9	-31.97	-19.44	7.59	Posterior
R10	*34.71*	*-23.23*	*12.18*		*L10*	*-32.00*	*-23.96*	*9.05*	

	Central Sulcus		
**Side**	**x**	**y**	**z**
**R**	35.29	-7.84	7.48
**L**	-32.76	-10.45	4.38

SUPPLEMENTARY TABLE 3. RETAINED BIPOLAR CHANNEL COUNTS BY INSULAR SUBREGION ACROSS MONITORING DAYS

Counts denote the number of retained bipolar channels within each insular quadrant (left/right × anterior/posterior) following preprocessing and quality-control procedures for each recording day

001

Day index	Left anterior	Left posterior	Right anterior	Right posterior	Total retained
1	0	0	1	2	3
2	0	0	2	3	5
3	0	0	0	3	3
4	0	0	1	1	2
5	0	0	1	1	2

002

Day index	Left anterior	Left posterior	Right anterior	Right posterior	Total retained
1	3	1	0	3	7
2	3	1	0	3	7
3	3	1	0	3	7
4	3	1	0	3	7
5	3	1	0	3	7
6	1	1	0	1	3

003

Day index	Left anterior	Left posterior	Right anterior	Right posterior	Total retained
1	4	2	2	3	11
2	4	2	2	0	8
3	4	2	2	2	10
4	0	0	0	0	0
5	4	2	2	1	9
6	4	1	0	0	5

004

Day index	Left anterior	Left posterior	Right anterior	Right posterior	Total retained
1	5	2	4	0	11
2	5	2	4	0	11
3	5	2	4	0	11
4	0	0	1	0	1
5	0	0	1	0	1

005

Day index	Left anterior	Left posterior	Right anterior	Right posterior	Total retained
1	4	1	4	1	10
2	4	1	4	1	10

Day index	Left anterior	Left posterior	Right anterior	Right posterior	Total retained
3	4	1	3	1	9
4	4	1	4	1	10
5	4	1	4	1	10
6	4	1	4	1	10
7	4	1	4	1	10

006

Day index	Left anterior	Left posterior	Right anterior	Right posterior	Total retained
1	5	2	5	2	14
2	5	2	5	2	14
3	5	2	5	2	14
4	5	2	5	2	14
5	1	2	3	2	8

SUPPLEMENTARY TABLE 4. DAILY CHRONIC PAIN AND CHRONIC PAIN-RELATED UNPLEASANTNESS RATINGS

Patient ID	Day	Pain intensity	Unpleasantness
001	1	10	9
001	2	9	8
001	3	6	5
001	4	4	3
001	5	4	3
002	1	8	7
002	2	6	6
002	3	4	4
002	4	2	1
002	5	1	1
002	6	2	1
003	1	10	9
003	2	9	8
003	3	8	8
003	4	6	5
003	5	5	4
003	6	5	4
004	1	6	7
004	2	4	5
004	3	5	4
004	4	5	4
004	5	2	2
005	1	5	8
005	2	6	7
005	3	8	6
005	4	7	4
005	5	6	3
005	6	7	2
005	7	2	2
006	1	5	7
006	2	4	6
006	3	6	5
006	4	3	4
006	5	4	3

Notes: Daily pain intensity and pain-related unpleasantness ratings were self-reported by participants immediately prior to each resting-state intracranial EEG recording. Ratings were provided on an 11-point numeric rating scale ranging from 0, indicating no pain or unpleasantness, to 10, indicating the worst imaginable pain or unpleasantness. These daily ratings were treated as continuous within-subject variables and entered as subject-mean–centered predictors in the mixed-effects models described in the Methods. Variability across days within each participant reflects natural day-to-day variation in chronic pain experience, rather than experimental manipulation.

SUPPLEMENTARY TABLE 5. Stable baseline spectral patterns of the human insula

Linear mixed-effects model results assessing stable anatomical organization of resting-state insular spectral features across insular quadrants. Analyses were performed on channel-day aggregated intracranial EEG data after quality control. Laterality is defined relative to each participant's chronic pain side. Models included Day_Index_c to account for slow temporal drift, with random intercepts for Subject and Channel nested within Subject. Partial R^2 reflects variance uniquely attributable to the specified fixed effect. **Model:** MeanVal ~ QuadRegion + Day_Index_c + (1 | Subject) + (1 | Subject:Channel); **Reference category:** Ipsilateral Anterior

A. Log-transformed spectral power (dB)

Band	Quadrant contrast	β	SE	*p*	Partial R^2	*q* (FDR)
Delta	Contralateral Posterior	+3.35	1.79	0.062	0.013	0.156
Delta	Ipsilateral Posterior	-3.04	2.34	0.19	0.007	0.415
Delta	Contralateral Anterior	+0.81	1.66	0.63	0.001	0.879
Theta	Ipsilateral Posterior	-4.65	2.23	0.038	0.017	0.114
Theta	Contralateral Posterior	+0.89	1.71	0.60	0.001	0.879
Theta	Contralateral Anterior	+0.14	1.59	0.93	<0.001	0.952
Alpha	Ipsilateral Posterior	-6.56	2.09	0.002	0.037	0.014
Alpha	Contralateral Posterior	-0.74	1.60	0.65	0.001	0.879
Alpha	Contralateral Anterior	-0.38	1.49	0.80	<0.001	0.923
Beta	Ipsilateral Posterior	-7.21	2.01	<0.001	0.047	0.006
Beta	Contralateral Posterior	-3.25	1.54	0.035	0.017	0.114
Beta	Contralateral Anterior	-0.48	1.43	0.74	<0.001	0.923
Gamma	Ipsilateral Posterior	-4.30	1.96	0.030	0.018	0.114
Gamma	Contralateral Posterior	-1.63	1.49	0.27	0.005	0.513
Gamma	Contralateral Anterior	+0.08	1.39	0.95	<0.001	0.952

B. Peak-derived and composite spectral metrics

Metric	Quadrant contrast	β	SE	p	Partial R^2	*q* FDR
Peak Dominant Freq (Hz)	Contralateral Posterior	-0.61	0.29	0.034	0.017	0.122
Peak Dominant Freq (Hz)	Ipsilateral Posterior	-0.68	0.37	0.065	0.013	0.145
Peak Dominant Freq (Hz)	Contralateral Anterior	-0.26	0.26	0.32	0.004	0.412
Peak Dominant Power (dB)	Ipsilateral Posterior	-4.88	2.30	0.034	0.017	0.122
Peak Dominant Power (dB)	Contralateral Posterior	+0.86	1.76	0.63	0.001	0.703
Peak Dominant Power (dB)	Contralateral Anterior	+0.08	1.64	0.96	<0.001	0.959
Slow Index	Contralateral Posterior	+0.96	0.47	0.041	0.016	0.122
Slow Index	Ipsilateral Posterior	+1.01	0.61	0.096	0.011	0.172
Slow Index	Contralateral Anterior	+0.44	0.43	0.31	0.004	0.412

Notes

- Quadrant contrasts are reported relative to ipsilateral anterior insula.
- β coefficients are unstandardized. For spectral power, β reflects differences in log-transformed spectral power expressed in dB, relative to the reference quadrant.
- Peak dominant frequency β values reflect Hz differences.
- The Slow Index was computed using linear spectral power, then $\log_{10}$-transformed for modelling; positive β values indicate relatively slower spectral composition.
- Partial R^2 values represent variance uniquely explained by the specified fixed effect after accounting for subject-, channel-, and day-level structure.
- P-values are nominal; FDR-adjusted q-values are reported.

SUPPLEMENTARY TABLE 6. POSTERIOR-RESTRICTED CONTRALATERAL DOMINANCE OF INSULAR SPECTRAL POWER.

Linear mixed-effects model results quantifying stable baseline hemispheric asymmetry in spectral power relative to the side of chronic pain. Analyses were performed on channel–day aggregated data.

Model Specification: Value ~ Laterality + Day_Index_c + (1|Subject) + (1|Subject:Channel).

Contrast: β coefficients reflect the "Contralateral minus Ipsilateral" difference. Positive values indicate that the contralateral (pain-processing) hemisphere maintains higher spectral power than the ipsilateral hemisphere

Band	Region	Contrast	β (dB)	SE	p	Partial R^2	q (FDR)
Delta	**Anterior**	Contra > Ipsi	~0	~0	ns	~0	ns
	Posterior	Contra > Ipsi	+8.52	2.42	<0.001	0.128	0.002
	Pooled	Contra > Ipsi	+2.51	1.40	0.074	0.014	0.37
Theta	**Anterior**	Contra > Ipsi	~0	~0	ns	~0	ns
	Posterior	Contra > Ipsi	+7.38	2.38	0.003	0.101	0.007
	Pooled	Contra > Ipsi	+1.79	1.34	0.18	0.005	0.45
Alpha	**Anterior**	Contra > Ipsi	~0	~0	ns	~0	ns
	Posterior	Contra > Ipsi	+7.15	1.81	<0.001	0.155	0.002
	Pooled	Contra > Ipsi	+1.53	1.28	0.23	0.002	0.45
Beta	**Anterior**	Contra > Ipsi	~0	~0	ns	~0	ns
	Posterior	Contra > Ipsi	+5.40	1.53	<0.001	0.128	0.002
	Pooled	Contra > Ipsi	+0.90	1.20	0.45	<0.001	0.56
Gamma	**Anterior**	Contra > Ipsi	~0	~0	ns	~0	ns
	Posterior	Contra > Ipsi	+3.67	1.50	0.016	0.066	0.032
	Pooled	Contra > Ipsi	+0.89	1.14	0.44	<0.001	0.56

Notes

- Positive β values indicate higher spectral power in the hemisphere contralateral to the chronic pain side.
- Contralateral dominance was observed predominantly in the posterior insula across multiple frequency bands, whereas anterior insula showed no consistent laterality effect.
- Models included Laterality and subject-centred Day_Index_c as fixed effects, with random intercepts for Subject and Channel nested within Subject.
- Partial R^2 indicates the proportion of variance uniquely attributable to laterality after accounting for subject, channel, and day-level structure.
- P values are nominal; q values reflect Benjamini-Hochberg false discovery rate correction applied within each regional family.
- Additional control analyses using anatomical hemisphere (left vs right) rather than pain-relative laterality did not identify fixed hemispheric asymmetry as an alternative explanation for these findings

SUPPLEMENTARY TABLE 7. Day-to-day associations between insular spectral power and pain intensity

Linear mixed-effects model results assessing within-subject day-to-day associations between daily pain intensity ratings and resting-state insular spectral power. Pain intensity was centred within subject to isolate day-level variation relative to each participant's own mean. Models included subject-level random intercepts and adjustment for longitudinal temporal drift. Partial R^2 reflects variance uniquely explained by pain intensity after accounting for subject-level baselines and day-level drift.

Log-transformed spectral power (dB)

Quadrant	Band	β	SE	p	Partial R^2	*q* (FDR)
Contralateral Posterior	Alpha	0.60	0.12	<0.001	0.330	<0.001
Contralateral Posterior	Beta	0.36	0.08	<0.001	0.295	<0.001
Contralateral Posterior	Gamma	0.22	0.06	0.001	0.182	0.014

Notes

- β coefficients represent unstandardized effects, expressed as change in log-transformed spectral power in dB per one-point increase in pain intensity.
- Pain intensity was centred within subject to isolate day-to-day variation and remove between-subject differences in baseline pain levels.
- Partial R^2 indicates the proportion of within-subject variance uniquely explained by pain intensity after accounting for subject-level baselines and longitudinal temporal drift.
- Only insular quadrants showing statistically significant pain-related associations are reported.
- In sensitivity analyses separating aperiodic and periodic spectral components, the pain-related alpha effect remained significant after removal of the fitted aperiodic component and after additional adjustment for aperiodic slope, supporting a genuine oscillatory contribution rather than a pure broadband slope effect.

SUPPLEMENTARY TABLE 8. Day-to-day associations between insular spectral power and pain unpleasantness

Linear mixed-effects model results assessing within-subject day-to-day associations between daily pain unpleasantness ratings and resting-state insular spectral power across insular quadrants. Pain unpleasantness was centred within subject to isolate day-level variation relative to each participant's own mean. Models included subject-level random intercepts and adjustment for longitudinal temporal drift. Partial R^2 reflects variance uniquely explained by pain unpleasantness after accounting for subject-level baselines and day-level drift.

Quadrant	Band	β	SE	p	Partial R^2	*q* (FDR)
Contralateral Posterior	Beta	+0.57	0.16	<0.001	0.194	0.027
Contralateral Posterior	Alpha	+0.64	0.28	0.024	0.095	0.166
Contralateral Posterior	Gamma	+0.32	0.13	0.016	0.107	0.166
Ipsilateral Anterior	Delta	+0.92	0.45	0.046	0.041	0.184
Ipsilateral Anterior	Gamma	+0.80	0.38	0.036	0.045	0.166

Notes

- β coefficients represent unstandardized effects, expressed as change in log-transformed spectral power in dB per one-point increase in pain unpleasantness.
- Pain unpleasantness was centred within subject to isolate day-to-day variation and remove between-subject differences in baseline unpleasantness.
- Partial R^2 indicates variance uniquely explained by pain unpleasantness after accounting for subject-level baselines and temporal drift.
- Only the contralateral posterior beta association survived false discovery rate correction; the remaining associations are nominal and are reported for completeness.
- In the aperiodic sensitivity analysis, the beta-unpleasantness relationship was attenuated after separation of aperiodic and periodic components and should therefore be interpreted more cautiously than the alpha-pain effect.

SUPPLEMENTARY TABLE 9. PEAK-DERIVED SPECTRAL METRICS AND DAY-TO-DAY PAIN-RELATED ASSOCIATIONS

Linear mixed-effects model results assessing stable anatomical differences and within-subject day-to-day associations for peak-derived spectral metrics. Analyses were performed on channel-day aggregated resting-state intracranial EEG data with explicit control for longitudinal temporal drift.

A. Stable baseline anatomical differences (anterior–posterior contrasts)

Comparison of posterior quadrants relative to the ipsilateral anterior insula (reference category).

Metric	Region (Contrast)	β	SE	p	Partial R^2	q (FDR)
Peak Dominant Freq (Hz)	Contralateral Posterior	-0.61	0.29	0.034	0.017	0.12
Peak Dominant Freq (Hz)	Ipsilateral Posterior	-0.68	0.37	0.065	0.013	0.15
Slow Index (log10)	Contralateral Posterior	+0.96	0.47	0.041	0.016	0.12
Slow Index (log10)	Ipsilateral Posterior	+1.01	0.61	0.096	0.011	0.17
Peak Dominant Power (dB)	Ipsilateral Posterior	-4.88	2.30	0.034	0.017	0.12

B. Day-to-day associations with pain intensity

Within-subject associations between daily pain intensity ratings and peak-derived spectral metrics.

Metric	Region	Predictor	β	SE	p	Partial R^2	q (FDR)
Peak Dominant Freq (Hz)	Contralateral Posterior	Pain Intensity	+0.12	0.04	0.008	0.01	0.024
Peak Dominant Power (dB)	Contralateral Posterior	Pain Intensity	+0.15	0.11	0.170	0.003	0.255
Slow Index (log10)	Contralateral Posterior	Pain Intensity	-0.01	0.02	0.650	<0.001	0.650

Notes

- Panel A: β coefficients represent stable differences relative to ipsilateral anterior insula.
- Negative β values for peak dominant frequency indicate lower dominant frequencies in posterior compared with anterior insula.
- Panel B: β coefficients represent the change in the specified metric per one-point increase in pain intensity, with pain intensity centred within subject.
- Partial R^2 indicates the proportion of variance uniquely explained by the specified fixed effect after accounting for subject, channel, and temporal structure.
- False discovery rate correction was applied within each panel across the listed metrics using the Benjamini-Hochberg procedure.
- The anatomical findings in this table describe stable within-cohort spectral organization and should not be interpreted as evidence for disease specificity in the absence of a pain-free invasive comparison group.

SUPPLEMENTARY TABLE 10. TEMPORAL DRIFT EFFECTS IN INSULAR SPECTRAL POWER

Linear mixed-effects model results assessing linear temporal drift in insular spectral power across the inpatient monitoring period. Analyses were performed on channel-day aggregated resting-state intracranial EEG data. Models included Day_Index_c as a fixed effect and random intercepts for Subject and Channel nested within Subject. Reported β coefficients represent change in spectral power per recording day.

Log-transformed spectral power (dB)

Region	Band	β (dB/day)	SE	p	Partial R²	FDR (q)
Contralateral Posterior	Theta	-0.77	0.12	<0.001	0.425	<0.001
Contralateral Posterior	Alpha	-0.60	0.14	<0.001	0.271	<0.001
Ipsilateral Posterior	Delta	-0.52	0.14	<0.001	0.316	0.003
Contralateral Anterior	Delta	-0.34	0.13	0.009	0.085	0.023
Contralateral Anterior	Theta	-0.32	0.12	0.009	0.085	0.023

Notes

- β coefficients represent unstandardized temporal drift, expressed as change in log-transformed spectral power in dB per recording day.
- Negative values indicate that spectral power decreased over the course of the monitoring period, consistent with signal settling, adaptation, or other non-specific longitudinal recording effects.
- Day_Index_c refers to gradual change across recording days within subject, not within-recording fluctuation.
- All pain-related analyses reported in Tables S7 to S9 included Day_Index_c as a covariate, ensuring that reported pain-physiology associations are statistically independent of this background temporal trend.
- P values are nominal; q values reflect Benjamini-Hochberg false discovery rate correction applied within this table.

SUPPLEMENTARY TABLE 11. Posterior-anterior organization of insular connectivity

Linear mixed-effects model results characterizing stable anatomical differences in intra-insular functional connectivity between posterior and anterior insular regions. Analyses were performed on pair-day aggregated daily means. Separate models were fitted for phase-locking value (PLV) and magnitude-squared coherence (COH) across canonical frequency bands. The fixed effect of Axis compares posterior against anterior insula, with anterior as the reference. Models included subject-centred Day_Index_c and random intercepts for Subject and Pair ID.

A. Phase-locking value (PLV)

Band	Posterior vs Anterior β	SE	p	q (FDR)	Partial R^2
**Delta**	+0.14	0.14	0.33	0.80	0.003
**Theta**	-0.13	0.19	0.49	0.80	0.002
**Alpha**	-0.07	0.21	0.75	0.80	<0.001
**Beta**	-0.06	0.23	0.80	0.80	<0.001
**Gamma**	+0.61	0.25	0.014	0.069	0.020

B. Coherence (COH)

Band	Posterior vs Anterior β	SE	p	q (FDR)	Partial R^2
**Delta**	0	0.03	0.97	0.97	<0.001
**Theta**	-0.04	0.04	0.30	0.76	0.004
**Alpha**	-0.03	0.04	0.47	0.79	0.002
**Beta**	-0.01	0.02	0.78	0.97	<0.001
**Gamma**	+0.06	0.02	0.002	0.008	0.033

Note

- β coefficients represent estimated differences in connectivity strength between posterior and anterior insula. Positive values indicate stronger connectivity in posterior insula.
- PLV was computed by band-pass filtering within canonical frequency bands, extracting the analytic phase with the Hilbert transform, and estimating the consistency of phase differences across time within each epoch.
- COH reflects magnitude-squared coherence averaged across the full canonical frequency band.
- Day_Index_c controls for gradual longitudinal change across recording days.
- False discovery rate correction was applied across the five frequency bands within each connectivity metric using the Benjamini-Hochberg procedure.
- Connectivity effect sizes were generally modest relative to local spectral power effects and are therefore interpreted as secondary rather than primary markers of day-to-day pain variation.
- Gamma-band PLV therefore reflects phase synchrony integrated across the full 30–55 Hz range rather than synchrony at a single peak frequency.

SUPPLEMENTARY TABLE 12. Frequency-dependent lateralization of insular connectivity relative to pain side

Linear mixed-effects model results assessing baseline hemispheric asymmetry in intra-insular functional connectivity relative to the chronic pain side. Analyses were performed on pair-day aggregated daily means. Separate models were fitted for phase-locking value (PLV) and magnitude-squared coherence (COH). Models included subject-centred Day_Index_c and random intercepts for Subject and Channel Pair.

A. Phase-locking value

Subregion	Band	Contrast	β	SE	p	q (FDR)
Anterior	Delta	Contra vs Ipsi	-0.15	0.12	0.24	0.59
Anterior	Theta	Contra vs Ipsi	-0.20	0.16	0.21	0.59
Anterior	Alpha	Contra vs Ipsi	-0.06	0.18	0.74	0.85
Anterior	Beta	Contra vs Ipsi	-0.17	0.20	0.40	0.85
Anterior	Gamma	Contra vs Ipsi	-0.02	0.17	0.90	0.94
Posterior	Delta	Contra vs Ipsi	+0.02	0.31	0.94	0.94
Posterior	Theta	Contra vs Ipsi	+0.18	0.40	0.65	0.85
Posterior	Alpha	Contra vs Ipsi	+0.24	0.44	0.59	0.85
Posterior	Beta	Contra vs Ipsi	-0.21	0.53	0.68	0.85
Posterior	Gamma	Contra vs Ipsi	-1.996	0.62	0.002	0.018
Long-Range	Gamma	Contra vs Ipsi	-0.506	0.15	<0.001	0.014

B. Coherence

Subregion	Band	Contrast	β	SE	p	q (FDR)
Anterior	Delta	Contra vs Ipsi	-0.033	0.03	0.22	0.66
Anterior	Theta	Contra vs Ipsi	-0.036	0.03	0.28	0.71
Anterior	Alpha	Contra vs Ipsi	-0.017	0.04	0.62	0.93
Anterior	Beta	Contra vs Ipsi	-0.008	0.02	0.72	0.94
Anterior	Gamma	Contra vs Ipsi	-0.002	0.01	0.85	0.94
Posterior	Delta	Contra vs Ipsi	-0.002	0.06	0.97	0.97
Posterior	Theta	Contra vs Ipsi	+0.009	0.06	0.87	0.94
Posterior	Alpha	Contra vs Ipsi	+0.042	0.08	0.58	0.93
Posterior	Beta	Contra vs Ipsi	-0.064	0.05	0.17	0.63
Posterior	Gamma	Contra vs Ipsi	-0.233	0.06	<0.001	0.010
Long-Range	Beta	Contra vs Ipsi	-0.010	0.005	0.038	0.29

Notes

- β coefficients represent the difference in connectivity strength for the contralateral hemisphere relative to the ipsilateral hemisphere. Negative β values indicate stronger ipsilateral connectivity.
- Day_Index_c controls for slow longitudinal signal change across recording days.
- False discovery rate correction was applied within each connectivity metric using the Benjamini-Hochberg procedure.
- Results demonstrate a selective ipsilateral dominance of gamma-band connectivity, most prominently in posterior and long-range pairings.
- Additional control analyses replacing pain-relative laterality with anatomical hemisphere did not support fixed left-right hemispheric asymmetry as an alternative explanation for the primary spectral findings

SUPPLEMENTARY TABLE 13. Within-subject associations between insular connectivity and daily pain ratings

Linear mixed-effects model results assessing within-subject day-to-day associations between daily pain ratings and insular functional connectivity. Analyses were performed on pair-day aggregated data. Separate models were fitted for pain intensity and pain unpleasantness to avoid multicollinearity. All models included subject-centred Day_Index_c and random intercepts for Subject and Channel Pair.

A. Pain intensity

Metric	Band	β	SE	p	q (FDR)	Partial R^2
PLV	Gamma	+0.080	0.023	0.001	0.002	0.013
PLV	Alpha	+0.019	0.005	<0.001	<0.001	0.017
PLV	Beta	+0.001	0.004	0.83	0.90	<0.001
PLV	Theta	-0.008	0.005	0.12	0.20	0.003
PLV	Delta	-0.001	0.005	0.90	0.90	<0.001
COH	Alpha	+0.003	0.001	<0.001	<0.001	0.019
COH	Gamma	-0.001	0.001	0.33	0.33	0.001
COH	Beta	-0.001	0.001	0.30	0.33	0.001
COH	Theta	-0.001	0.001	0.12	0.31	0.003
COH	Delta	-0.001	0.001	0.24	0.33	0.002

B. Pain unpleasantness

Metric	Band	β	SE	p	q (FDR)	Partial R^2
PLV	Alpha	+0.047	0.013	<0.001	0.002	0.013
PLV	Gamma	+0.100	0.064	0.12	0.20	0.003
PLV	Beta	-0.018	0.010	0.08	0.19	0.003
PLV	Theta	+0.013	0.013	0.34	0.34	0.001
PLV	Delta	+0.015	0.013	0.26	0.32	0.001
COH	Alpha	+0.003	0.002	0.25	0.53	0.001
COH	Gamma	+0.003	0.003	0.32	0.53	0.001
COH	Beta	<0.001	0.002	0.83	0.90	<0.001
COH	Theta	+0.005	0.002	0.046	0.23	0.004
COH	Delta	<0.001	0.003	0.90	0.90	<0.001

Notes

- Model specification: Connectivity ~ Predictor_c + Day_Index_c + (1 | Subject) + (1 | Subject:Channel_Pair).
- Behavioural predictors were centred within subject to isolate day-to-day associations and remove between-subject differences.
- Day_Index_c controls for subject-specific longitudinal signal drift across recording days.
- Partial R^2 reflects variance uniquely attributable to the pain rating after accounting for random effects and temporal drift.
- False discovery rate correction used the Benjamini-Hochberg procedure within each Predictor × Metric family across frequency bands.
- Pain-related associations with insular connectivity were detectable but small in magnitude, and substantially weaker than corresponding spectral power effects reported in Tables S7 to S9.
- These findings should therefore be interpreted cautiously and as supportive secondary observations rather than dominant drivers of day-to-day pain-related variation.

SUPPLEMENTARY TABLE 14. Combined models of insular connectivity including both pain dimensions

Linear mixed-effects model results assessing within-subject day-to-day associations between daily pain intensity, pain unpleasantness, and insular functional connectivity when both pain dimensions were included simultaneously. Analyses were performed on pair-day aggregated data to evaluate the robustness of connectivity associations under a more conservative model specification. Anatomical specificity was explicitly modeled using insular Axis and Laterality.

A. Pain intensity

Metric	Band	Axis	Laterality	β	q (FDR)	Partial R^2
COH	Alpha	Anterior	Ipsilateral	+0.006	< 0.05	0.027
COH	Beta	Posterior	Ipsilateral	+0.027	< 0.05	0.029
PLV	Theta	Anterior	Contralateral	−0.010	< 0.05	0.028

B. Pain unpleasantness

No associations survived false discovery rate correction in any frequency band or anatomical subdivision when pain intensity was included in the same model.

Notes

- **Model specification:** Connectivity ~ PainIntensity_c + Unpleasantness_c + Axis × Laterality + Day_Index_c + (1 | Subject) + (1 | Subject:Channel_Pair)
- Behavioural predictors were centred within subject to isolate day-to-day associations and remove between-subject differences.
- Day_Index_c was included to account for slow longitudinal signal drift across the monitoring period.
- Random intercepts for Subject and Channel Pair account for repeated measures and pair-specific baseline connectivity.
- Partial R^2 reflects variance uniquely attributable to the specified behavioural predictor after accounting for random effects and temporal drift.
- False discovery rate correction was applied within each Predictor × Metric family across frequency bands and anatomical cells.
- Effect sizes in combined connectivity models were uniformly small, consistent with the broader conclusion that insular connectivity is comparatively stable and that pain-related variance is more robustly expressed in local spectral power than in pairwise connectivity measures.
